# Eye Selector Logic for a Coordinated Cell Cycle Exit

**DOI:** 10.1371/journal.pgen.1004981

**Published:** 2015-02-19

**Authors:** Carla S. Lopes, Fernando Casares

**Affiliations:** CABD (Andalusian Centre for Developmental Biology), C.S.I.C.-Universidad Pablo de Olavide-Junta de Andalucía, Seville, Spain; New York University, UNITED STATES

## Abstract

Organ-selector transcription factors control simultaneously cell differentiation and proliferation, ensuring the development of functional organs and their homeostasis. How this is achieved at the molecular level is still unclear. Here we have investigated how the transcriptional pulse of *string/cdc25 (stg)*, the universal mitotic trigger, is regulated during *Drosophila* retina development as an example of coordinated deployment of differentiation and proliferation programs. We identify the eye specific *stg* enhancer, *stg*-FMW, and show that Pax6 selector genes, in cooperation with Eya and So, two members of the retinal determination network, activate stg-*FMW*, establishing a positive feed-forward loop. This loop is negatively modulated by the Meis1 protein, Hth. This regulatory logic is reminiscent of that controlling the expression of differentiation transcription factors. Our work shows that subjecting transcription factors and key cell cycle regulators to the same regulatory logic ensures the coupling between differentiation and proliferation programs during organ development.

## Author Summary

Organs develop from groups of undifferentiated cells that proliferate and differentiate into specific cell types. During development, the coupling between proliferation and differentiation programs ensures that enough cells of the different cell types are generated. This is critical for proper organ formation and function. Here, we use the developing *Drosophila* eye to examine how the coupling between these two programs is achieved. During eye development, progenitors are amplified before they exit the cell cycle and enter the differentiation program. This amplification step depends on an expression burst of the mitotic trigger *string/cdc25*, which, by forcing cells into mitosis, synchronizes cells in G1 just before differentiation onset. Thus *string* regulation acts as a hub where differentiation and proliferation programs are integrated. We identify a DNA element that controls the burst of *string* expression prior to differentiation, and show that it is regulated by the same gene network that triggers eye development. The transcription factor Pax6/Eyeless is a key regulator in this network. Eyeless acts cooperatively with Sine oculis and Eyes absent to regulate *string*, through a positive feed-forward loop. This loop is negatively modulated by the progenitor-specific transcription factor Homothorax/Meis1. This work shows that transcription factors that instruct cells to acquire an eye fate also control their proliferation regime, thus guaranteeing the coupling between proliferation and differentiation.

## Introduction

Selector genes are transcription factors that instruct the development of organs. The processes under the control of selector genes include the assignation of cell fates and their organ-specific responses to extracellular signals [1]. But organ development also requires the faithful execution of proliferation programs to ensure the expansion of progenitor cells and their coordinated exit from the cell cycle prior to the onset of differentiation. This coordinated cell cycle exit is critical to regulate organ size during development and to ensure tissue homeostasis during adult life. The power of selector genes to control the differentiation state of cells and their proliferation regimes explains why abnormal expression of these transcription factors is often associated to cancer [reviewed in 2,3]. However, how selector genes carry out the coordination between proliferation and differentiation programs is still unclear.

Structures of the nervous system, such as the retina, in which complex arrays of different cell types need to be assembled from multipotent proliferative progenitors, are especially sensitive to impairments of proliferation control mechanisms [reviewed in 4,5]. It is therefore likely that selector genes coordinate cell cycle exit with the processes of differentiation and patterning by co-regulating the transcription of cell cycle and patterning genes. However, this control may be direct or mediated by intermediate transcription factors.

The eye selector function is exerted by a network of transcription factors and signaling pathways, with many of the network genes shared by invertebrates and vertebrates. The Pax6 selector genes are on top of the retinal determination (RD) gene network in both animal groups [6]. Pax6 mutations are responsible for aniridia [7,8], while Pax6 overexpression is associated with retinoblastoma cancer progression through promotion of proliferation and cell survival [9–11].

In *Drosophila*, the RD gene network comprises a number of transcription factors and nuclear proteins, that includes members of conserved gene families: The Pax6 paralogues *eyeless (ey)* and *twin of eyeless (toy)*; the Six family genes *Optix (*Six3) and *sine-oculis (so*; Six1,2); So’s partner, *eyes absent (eya); dachshund (dac*); and the Meis1 homologue *homothorax (hth)*. These genes are not only connected through transcriptional cross-regulation, but also have been found to engage in protein complexes [reviewed in 12,13].

Research during the past years is yielding an increasingly clearer picture of how the process of eye specification and retinal patterning in *Drosophila* is controlled [reviewed in 13,14]. The eye primordium (also called “eye disc”) derives from the So-expressing embryonic cephalic neuroectoderm [15]. Within this domain, *toy* activates *ey* expression during late embryogenesis, which results in the specification of the eye-progenitor cells [16]. During larval life, *ey-*expressing progenitors are maintained proliferative and multipotent as long as they express *hth* [17–19]. Repression of *hth* starts during the third and last larval stage (L3), mediated by Decapentaplegic (Dpp a BMP2/4-like molecule) and Hedgehog (Hh) signals produced at a moving signaling center, called “morphogenetic furrow” (MF). *hth* repression is key, as it allows the upregulation of *so*, *eya* and *dac* [17,19]. Coinciding with *hth* repression, the expression of *string (stg)-*the *Drosophila cdc25* phosphatase homologue [20–22]- is upregulated and this drives cells through a few consecutive mitotic rounds (the first mitotic wave, FMW), resulting in G1-synchronized *ey*-*so-eya-dac-*expressing cells (retinal precursors) [17,19,20]. The expression of *ey*, *so* and *eya* turns on the expression of the bHLH gene *atonal (ato)*, the fly homologue of *ath5/atoh7*, which is necessary for the differentiation of precursor cells into photoreceptors, lens and pigment cells of the retina [23–26].

The information processing devices in networks such as the RD gene network are *cis*-regulatory elements (CREs), DNA sequences that allow binding of specific combinations of transcription factors, which in turn regulate transcription of the CRE target genes [27]. Therefore, CREs are key to understand the logic that drives the developmental processes directed by a gene network. In the *Drosophila* RD gene network, CREs from *ey* [28,29], *so* [30,31], *eya* [32], *dac* [33]; *optix* [34] and *ato* [24–26,35] have been isolated and studied in molecular detail. Not surprisingly, all rely on direct Pax6 input and at least *so*, *dac* and *ato* CREs also integrate direct regulation by the So:Eya complex [24–26,31,33]. But all of these genes are transcription factors, not effector genes. Is the logic acting upon transcription factors the same as that controlling specific outputs of the network’s function—such as cell cycle control?

In this paper we have addressed this issue by investigating the direct regulatory logic acting upon the eye-specific *stg* CRE. During *Drosophila* retina development, a transcriptional burst of *stg* is associated to the transition from proliferative progenitors to cell cycle quiescent precursors [19,20,22]. This peak of *stg* drives progenitors, which are mostly in the G2 phase of their cell cycle, through the FMW, leading to their G1 synchronization [19,36]. This synchronicity is essential: In *stg* ^*hwy*^ mutants, which lack specifically this peak of *stg* expression, precursors are specified but do not become G1-synchronized. As a result, the patterning of the retina is aberrant [22]. Therefore, the study of *stg* transcriptional regulation in the eye offers an ideal model to understand how organ specific cell cycle and patterning programs are coupled during development.

We identified a distal 5′*stg* CRE, which we named *stg-FMW* (First Mitotic Wave) enhancer. When *stg* expression was driven by *stg-FMW* enhancer, it rescued the eye defects of *stg* ^*hwy*^ mutants, indicating that *stg-FMW* contains most, if not all the regulatory information required for the accurate spatial-temporal expression of *stg* at the progenitor-precursor transition. Within this element, we characterized two positive inputs: one from both Pax6 proteins, Ey and Toy, and one from So:Eya. Interaction with these transcription factors occurs through two binding sites. We also identified one negative input: Hth. In agreement, assays *in vivo* suggested that Hth hampers Ey activation of *stg-FMW*. This fact could explain mechanistically the negative action of Hth on *stg* transcription. The picture that emerges is of a coherent feed-forward loop in which Ey and Toy play partially redundant activating roles, together with So:Eya, on *stg* transcription. Moreover, this activation is modulated by the negative input of the *meis1* gene, *hth*.

## Results

### Identification of eye-specific *stg* CREs

**Fig 1:**
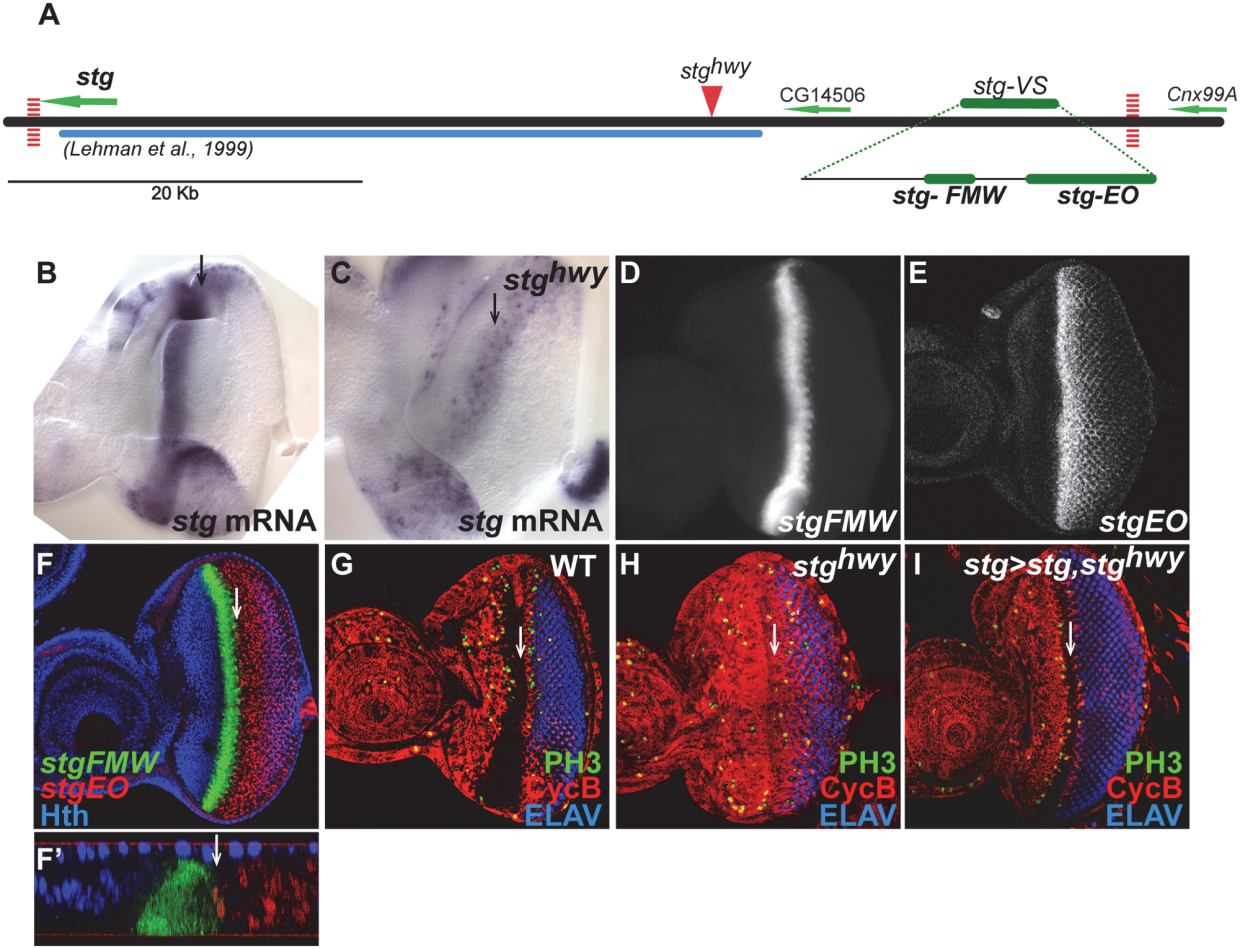
Identification of eye-specific *stg* CREs. (A) Map of the *stg* genomic locus, covering a region of ~62 Kb, which includes CG14506. Green arrows delimit the coding regions and indicate direction of transcription. Vertical dashed red lines map the position of CTCF binding sites downstream of *stg* and *Cnx99A*. Solid blue line delimits the region previous screened by Lehman et al. [21]. Solid red arrowhead indicates the insertion point of the gypsy transposon in *stg* ^*hwy*^ allele. Solid green lines indicate the position of the CREs that showed expression in the visual system, *stg-VS*, *stg-FMW* and *stg-EO*. (B, C) Expression of *stg* mRNA in wild type and *stg* ^*hwy*^ eye imaginal discs. (B) Expression can be detected in the precursor cells domain, anterior to the MF and ocellar domain. (C) In *stg* ^*hwy*^ homozygous eye imaginal discs expression can only be detected posterior to the MF. (D, E) Expression of dGFP driven by *stg*-FMW and *stg*-EO enhancer fragments. (F) Eye imaginal disc stained for *stg*-FMW (green), *stg*-EO (red), and Hth (blue). *stg*-FMW and *stg*-EO are expressed in non-overlapping domains. *stg*-FMW drives expression in precursor cells and abuts the domain of Hth expression (blue). *stg*-EO drives expression in cells posterior to the MF. (F’) Cross-section from an inset of F, showing that *stg*-FMW and *stg*-EO patterns do not overlap. (G-H) Wild-type (G) and *stg* ^*hwy*^ homozygous (H) L3 eye imaginal discs stained for the G2 marker Cyclin B (red), the mitotic marker phospho-Histone H3 (PH3; green) and the photoreceptor marker ELAV (blue). (I) *stg* ^*hwy*^ homozygous disc ectopically expressing Stg, driven by *stgFMW*-Gal4, and stained for cyclin B (red), PH3 (green) and ELAV (blue). Ectopic expression of Stg in the *stg*-FMW expression domain restores the pattern of Cyclin B (red) and mitosis (green) to wild type. In all images anterior is to the left. White arrow indicates the MF.

As an entry point into the molecular mechanisms by which selector transcription factors activate organ-specific programs of cell division, we searched for an eye specific regulatory element of *stg*. Lehman and co-workers had scanned 38 Kb of the *stg* locus (from-35 to +3 relative to the transcription start site) and uncovered several CREs [21 and Fig. 1A]. These included CREs active in embryos and imaginal discs, but none of the fragments studied recapitulated the strong stripe of *stg* expression anterior to the MF (Fig. 1B). We re-analyzed a similar interval of 38.7 Kb (from the *stg* transcription start site [Chr3R: 25.081.410] to CG14506 [Chr3R: 25.120.100], the gene located immediately upstream of *stg*) by generating a new set of tiled reporter transgenes with an average fragments length of 5 Kb. Contiguous fragments overlap each other an average length of 1,5 Kb (Fig. 1A). This approach was selected to avoid splitting blocks of conserved sequence, as sequence conservation is often a landmark of CREs [37]. Again, none of the fragments from this interval revealed an expression pattern reminiscent of *stg* in the eye disc.

Together with the Lehman study, our results suggested that the eye-specific CREs should be located further upstream [21]. To try to define the expected limit of the *stg* regulatory landscape we used several landmarks. First, the analysis of an extended genomic region revealed the existence of two class I insulator binding sites [38,39], one immediately downstream of the *stg* transcript (Chr3R: 25077239) and another downstream of *Cnx*99A (Chr3R: 25138877), delimiting a region of 61,6 Kb (Fig. 1A). Binding of class I insulators helps to establish chromatin boundaries between genes [39,40]. Therefore, we considered that this region might comprise the *stg* regulatory landscape and should include unidentified *stg* CREs. This interval includes CG14506 as well. However, this transcript is not conserved in all *Drosophila* species sequenced, although the adjacent sequences are highly conserved, suggesting that CG14506 is a bystander gene within the *stg* locus. Second, a regulatory mutation in the *stg* gene, *highway (stg^hwy^)*, had been shown to be associated to an insertion of an uncharacterized DNA sequence at around 30 Kb upstream of the *stg* transcription start site. The *stg* ^*hwy*^ is a viable allele that results in slightly reduced, roughened eyes [22]. In *stg* ^*hwy*^ mutant eye discs the peak of *stg* expression at the progenitor-precursor transition is lost (Fig. 1B, C). As a consequence, cells fail to undergo G1 arrest, and accumulate in G2, with high levels of mitotic cyclins, such as cyclin B [Fig. 1G, H and 22]. Since *stg* ^*hwy*^ is an eye-specific regulatory allele of *stg*, we reasoned that the *stg* ^*hwy*^ insertion might be affecting the CRE we were looking after, perhaps having landed in its vicinity.

A primer walking strategy was next employed to identify the nature of the DNA element and the exact insertion point in *stg* ^*hwy*^. Molecularly, we defined the mutation associated with *stg* ^*hwy*^ as an insertion of a gypsy transposable element between positions Chr3R: 25115094 and Chr3R: 25115097 (Fig. 1A and S1A Fig.). Gypsy transposable elements are known to block enhancer-promoter interactions when located in between them [reviewed in 41]. This finding suggested that the insertion in *stg* ^*hwy*^ was likely impairing the contacts between the eye-specific CRE and the *stg* promoter. Further, it predicted that the eye CREs should lie between the genes CG14506 and *Cnx*99A. When we extended our reporter transgene study to this region, we identified a fragment of 4.8 Kb, located distal to CG14506 and 52 Kb away from the *stg* promoter. This fragment was sufficient to drive expression of the reporter gene (destabilized Green Fluorescent Protein, (dGFP)) in eye discs, both in a stripe anterior to the MF as well as in cells posterior to it (Fig. 1A and S1B Fig.). In addition, this fragment showed enhancer activity in the dorsal anterior region of the eye disc, where the prospective ocellar region resides, and in the lamina region of the optic lobes. We named it *stg-VisualSystem* (*stg-VS*).

We next subdivided *stg-VS* into smaller overlapping fragments. This allowed the identification of two enhancer elements, of 539bp and 690bp respectively, that drive expression in different cell populations of the eye disc (Fig. 1A, D, E). The remaining sub-fragments of *stg-VS* failed to drive expression in the visual system or elsewhere. The 539bp enhancer drives strong dGFP expression in the FMW domain and precursor cells and recapitulates *stg* expression in the eye field after differentiation onset (S2 Fig.). Accordingly, the 539bp enhancer was called *stg-First Mitotic Wave* (*stg-FMW*) (Fig. 1D). The 690bp element drives expression in the ocellar domain, and in a subset of cells posterior to the MF. This fragment was named *stg-EyeOcelli* (*stg-EO*) (Fig. 1E). *stg-FMW* and *stg-EO* are expressed in adjacent, non-overlapping domains (Fig. 1F, F’) and together reconstitute the eye disc-specific pattern of *stg* transcription. Anteriorly, expression driven by *stg-FMW* abuts the Hth expression domain (Fig. 1F, F’), as was previously shown for *stg* mRNA [19]. Expression of *stg-EO* in the eye field overlaps the so-called second mitotic wave [SMW, 42]. To test that *stg-FMW* is a functional *stg* enhancer, we attempted to rescue the *stg* ^*hwy*^ phenotype, by driving *stg* expression using a *stg-FMW-GAL4* driver in *stg* ^*hwy*^ homozygous individuals. *stg-FMW-GAL4>UAS-stg* rescued the adult eye phenotype and the pattern of cyclin B accumulation in L3 eye discs of *stg* ^*hwy*^ mutants (Fig. 1G-I). This result supports the idea that *stg-FMW* is a functional, eye-specific *stg* CRE and, together with the data on enhancer activity throughout the *stg* locus, suggests that it may be the sole CRE responsible for *stg* expression at the FMW.

### Ey and Toy are redundantly required to activate *stg-FMW*

**Fig 2:**
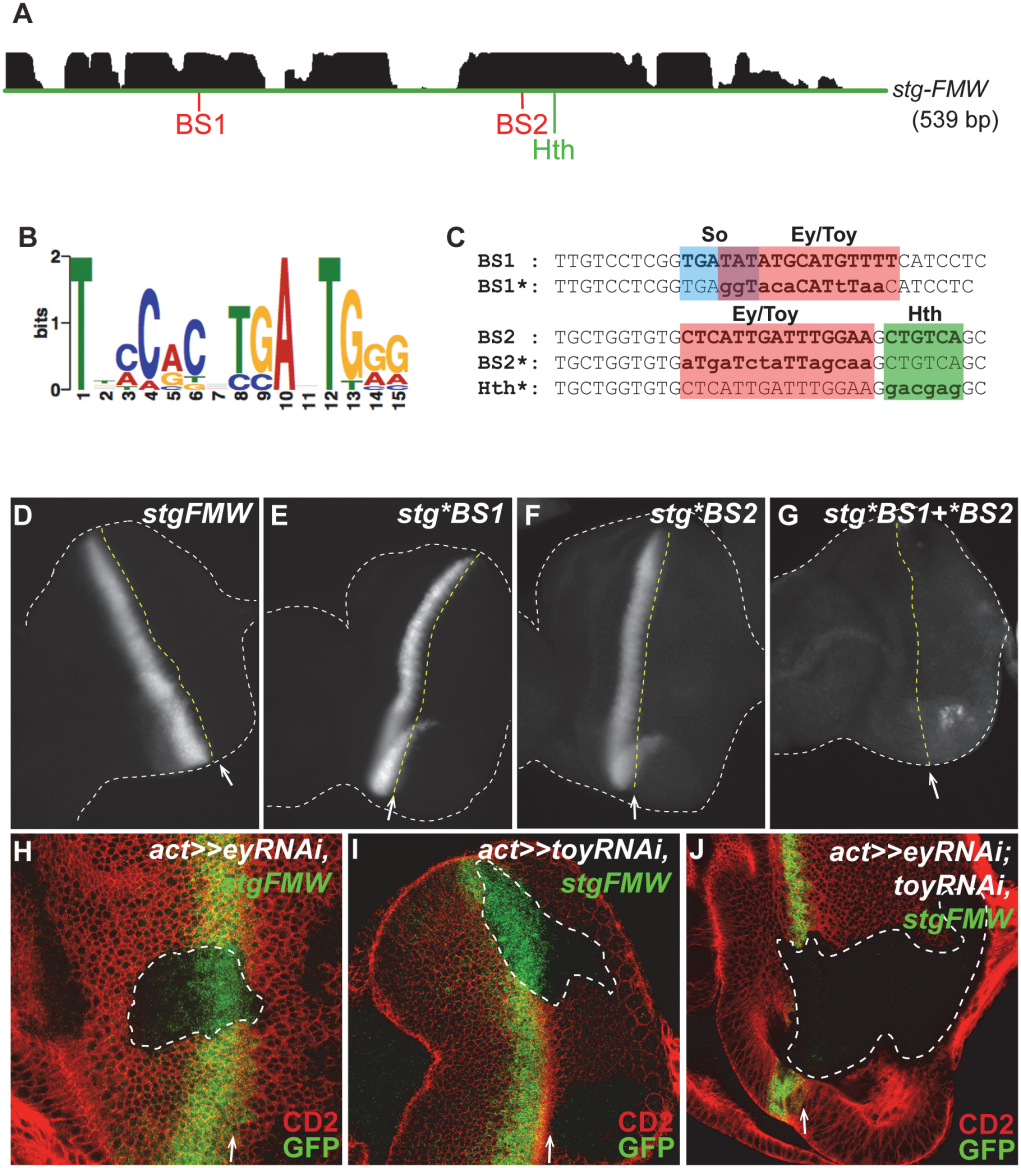
Ey and Toy are redundantly required to activate *stg-FMW*. (A) The pattern of conservation of *stg*-FMW enhancer sequence in *Drosophila* species as displayed by the UCSC genome browser (http://genome.ucsc.edu) is shown at the top. Two highly conserved regions harboring binding sites for Pax6, BS1 and BS2, and Hth are shown below. (B) Logo of the optimized PAX6 Position Weight Matrix (PWM) used in this study. Nucleotide preference is represented by the height of the letter. (C) Partial sequence of BS1 and BS2. BS1 contains binding sites for So and Pax6 genes (Ey/Toy) highlighted in blue and red, respectively. BS1*: Mutated version of BS1. Ey/Pax6 mutated bases are shown in lowercase. BS2 contains adjacent binding sites for Pax6 (Ey/Toy) and Hth, highlighted in red and green respectively. Mutated core bases for Pax6 (Ey/Toy) and Hth are shown in lowercase in BS2*. (D—G) Analysis of the expression of *stg*-FMW upon mutation of BS1 or BS2. Representative L3 eye imaginal discs carrying the wild type *stg-FMW* (D), and the mutated versions stg*BS1 (E) and stg*BS2 (F) of the enhancer. (G) Expression of GFP is not affected upon mutation of BS1 or BS2 in *stg*-FMW. Simultaneous mutation of BS1 and BS2 (stg*BS1+*BS2) abolishes GFP expression in the eye field; small GFP dots can be detected but only at the dorsal and ventral margins of the eye disc. Discs are outlined by the white dashed lines. The position of the MF is indicated by the white arrow and yellow dashed line. (H—J) Clones of *ey* (H) or *toy* (I) RNAi, in the eye imaginal disc. Clones are outlined and marked by the absence of CD2 (red). Expression of *stg-FMW* (green) is not affected by downregulation of Ey or Toy. (J) Eye disc containing *ey* and *toy* double mutant clones, stained for CD2 (red) and *stg-FMW* (green). In clones of cells double mutant for *ey* and *toy* the expression of *stg-FMW* is lost. White arrow indicates the position of the MF. In all images anterior is to the left.

The *stg*-FMW sequence shows a high degree of conservation (Fig. 2A). Using JASPAR and TRANSFAC models [43,44] we predicted the existence of putative transcription factor binding sites for components of the RD network. For the identification of Ey binding sites we generated our own position weight matrix from a set of published binding sites [24,30,34,45] (Fig. 2B and S3 Fig.). Evolutionarily conserved Ey binding sites in the genome of 12 *Drosophila* species were filtered using the CBS platform [46]. Two highly conserved regions were identified, which we refer to as Binding Site 1 (BS1) and BS2 (Fig. 2A, C). BS1 contains partially overlapping putative binding sites for Ey/Pax6 and So (Fig. 2C). BS2 contains one Ey/Pax6 conserved binding site, and a highly conserved consensus site for Hth lies adjacent to it (Fig. 2A, C).

To test the *in vivo* relevance of BS1 and BS2, we mutated the bases fitting the Ey/Pax6 consensus at each site. Transgenic lines carrying mutant versions of the *stg-FMW* enhancer harboring mutations in BS1 (stg*BS1), in BS2 (stg*BS2) or in both sites (stg *BS1+*BS2) were analyzed (Fig. 2D-G). Neither the stg*BS1 nor the stg*BS2 single mutants showed altered temporal or spatial expression (Fig. 2E, F), suggesting that the remaining site suffices for enhancer activity during eye development. However, when the two sites were simultaneously mutated (stg*BS1+*BS2), enhancer activity was lost (Fig. 2G). This shows that both sites are redundant for enhancer activity *in vivo*.

Since mutation of both Ey/Pax6 consensus-binding sites abolished enhancer activity, we next assayed whether Ey was required for *stg-FMW* activity. The expression of *stg-FMW* remains unaffected in clones where *ey* expression has been knocked-down using RNAi (Fig. 2H). As *toy*, a second Pax6 gene, is expressed coextensively with *ey* in the eye primordium [16], we tested if Toy was required for regulation of *stg-FMW*. As observed with *ey*, *toy* downregulation through RNAi did not affect enhancer activity (Fig. 2I).

Since Ey and Toy have similar expression patterns and binding site preferences [16,31,47], we next tested a potential redundant function of Ey and Toy in *stg-FMW* regulation. For this, we generated clones of cells in which both genes were simultaneously knocked-down by co-expression of *ey-RNAi* and *toy-RNAi*. In these cells the activity of *stg-FMW* was abolished in a cell-autonomous way (Fig. 2J). This result shows that both Ey and Toy redundantly activate the *stg-FMW* enhancer. A redundant function between Ey and Toy was further supported by the finding that in *ey* ^*2*^ homozygous imaginal discs, where *toy* expression is maintained [16], the pattern and levels of expression of *stg* mRNA or *stg*-FMW were not affected (S4A-G Fig.).

To explore a potential “division of labor” between the two sites, with each of them specializing in only one of the two Pax6, we generated clones of *ey*-RNAi and *toy-*RNAi in the presence of *stg*-*FMW* mutated versions (stg*BS1 and stg*BS2) (S4H-K Fig.) Upon mutation of BS1 or BS2, downregulation of Ey did not abolish enhancer activity (S4F,G Fig.). Downregulation of Toy did not impact on the activity of the single-mutant versions of *stg-FMW* either (S4J, K Fig.). These results show that one Ey/Pax6 binding site suffices for enhancer activity, and that Ey and Toy do not have preferential binding *in vivo*.

### Eya/So cooperate with Ey/Toy and act as positive regulators of *stg-FMW*

**Fig 3:**
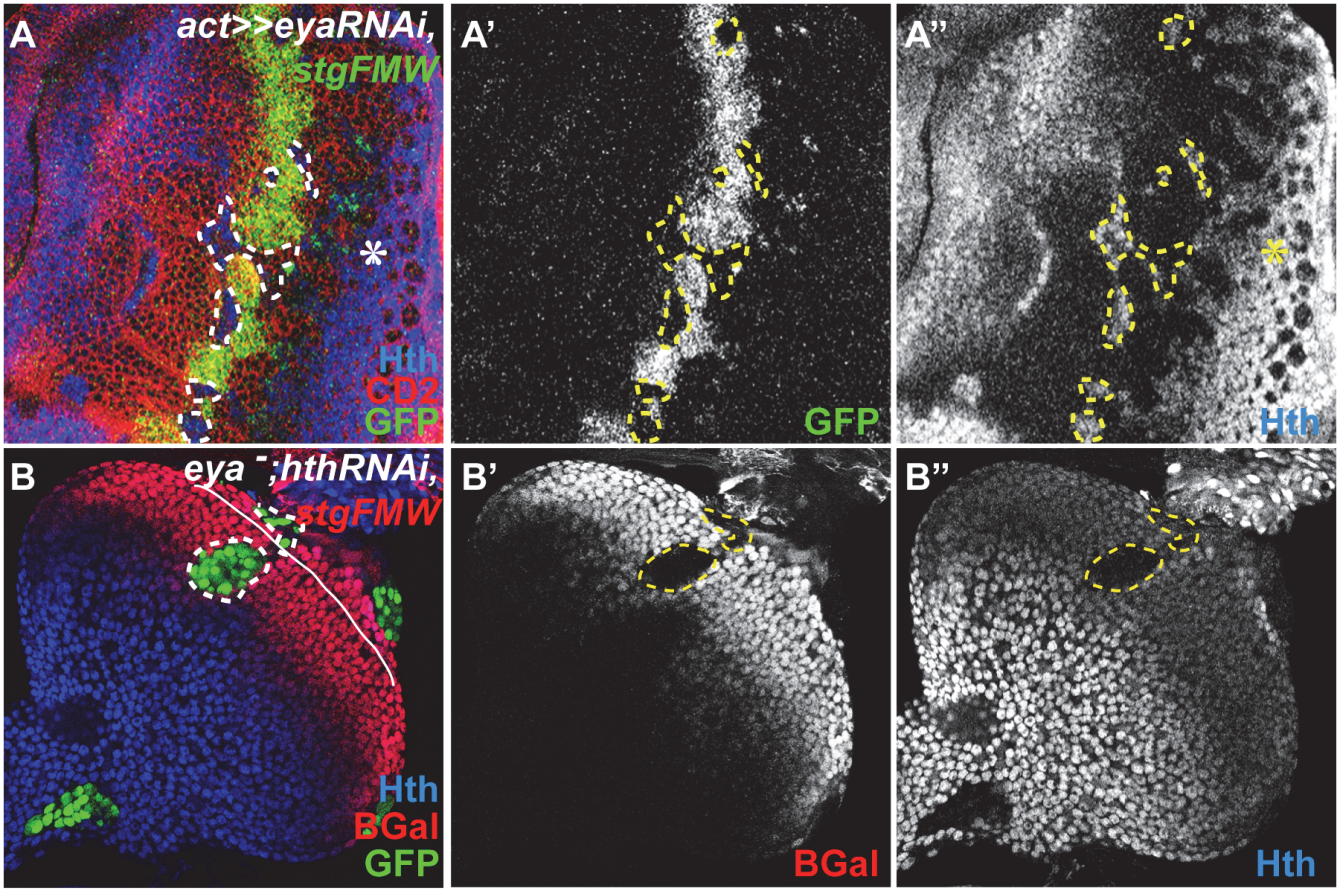
So/Eya are positive regulators of *stg-FMW*. (A) Eye imaginal disc with clones of cells mutant for *eya*, labeled by the absence of CD2 (red). (A’) Within the clone cells, *stg-FMW* expression (green) is not activated. (A”) Hth expression (blue) is maintained in *eya* RNAi cells. (B) MARCM *eya^-^hth^-^* clones (GFP) stained for *stg-FMW* (lacZ- red) (B’) and Hth (B”)(blue), showing that *eya* is required for *stg-FMW* expression. Clones are outlined. The asterisk (_*_) in A indicates the normal expression pattern of Hth, during L3, in pigment progenitor cells posterior to the MF [17]. In all images anterior is to the left.

Region BS1 also contains a putative binding site for So (Fig. 2C). So is known to physically interact with the transcriptional co-activator Eya to regulate downstream genes [48]. We next tested the role of So and its transcriptional co-activator Eya in the regulation of *stg-FMW*. Both genes lay downstream of Ey in the RD gene network [reviewed in 14,49] and *stg* has been previously identified as a transcriptional target of the Eya:So complex [50]. Loss of function of Eya (S5A Fig.) or its downregulation by means of RNAi (Fig. 3A) in cell clones resulted in a cell-autonomous loss of enhancer activity in the precursor domain. A similar result was obtained when So expression was knocked down using RNAi (S5B Fig.). This loss of enhancer activity coincided with the maintenance of high levels of Hth expression (Fig. 3A” and S5B Fig.).

**Fig 4:**
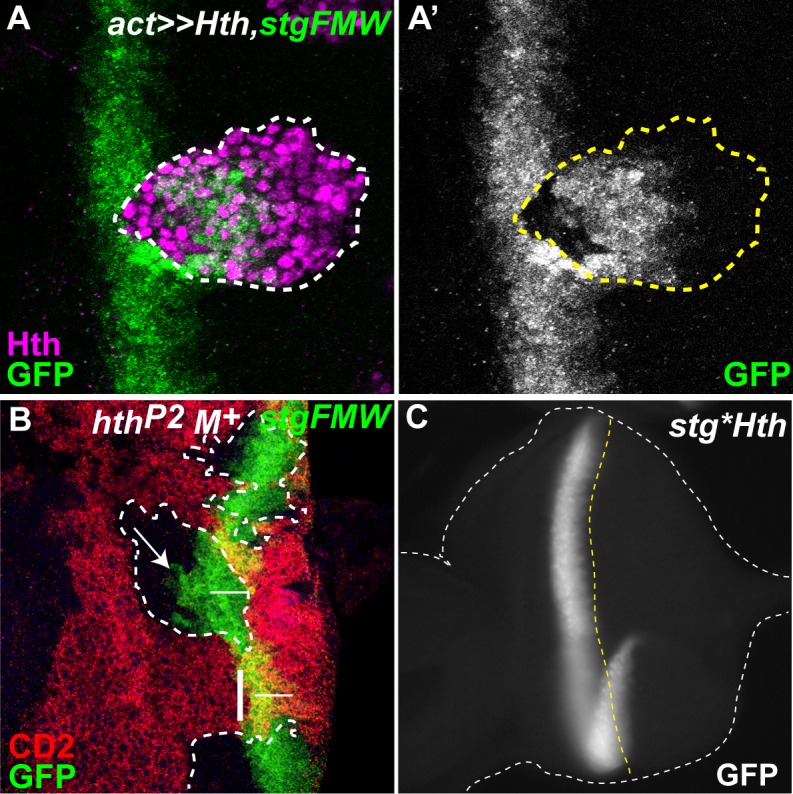
Hth defines the anterior domain of *stg-FMW* expression. (A) Clone of cells ectopically expressing Hth-HA, labeled in magenta, and stained for *stg*-*FMW* (green). (A’) Expression of *stg-FMW* is only repressed within the anterior domain of the clone. (B) Eye disc containing clones of a null allele of *hth* (*hth* ^*P2*^), stained for CD2 (red) and *stg-FMW* (green). Clone cells are labeled by the absence of CD2. Within the clone *stg-FMW* expression occurs in a few cell rows anterior to its normal domain of expression. Arrow indicates premature expression of GFP. The vertical dashed line limits the most anterior expression of *stg*-FMW in wild-type tissue. The horizontal line represents the width of *stg*-FWW expression in wild-type tissue. (C) Mutation of the Hth BS (stg*Hth) does not affect the spatial or temporal expression of *stg-FMW*.

We had previously shown that Hth could act as a repressor of *stg* transcription [19]. Additionally, Eya:So are negative regulators of Hth expression during eye development [17]. Therefore, the observed loss of *stg-FMW* enhancer activity could result from either the loss of Hth repression, or alternatively reflect a positive requirement of Eya:So for *stg-FMW* activation. To discriminate between these two hypotheses, we first checked whether ectopic expression of Hth could repress *stg-FMW*. In Hth-expressing clones *stg-FMW* activation was delayed, but not repressed (Fig. 4A). These findings were qualitatively different from the ones obtained upon RNAi-mediated *eya* knock-down, where loss of enhancer activity was always observed, irrespective of the position of the clones within the precursor cell domain. This suggested that indeed Eya:So acted as *stg-FMW* activators. To test this issue avoiding any interference by Hth, we generated clones of cells simultaneously mutant for *eya* (*eya* null) and *hth* (*hth* RNAi), using the MARCM system (Fig. 3B) [51]. In *eya- hth-* cells, *stg-FMW* activity was always lost in a cell-autonomous manner. However, in these *eya- hth-* double mutant cells expression of Ey was maintained (S5C Fig.). Therefore, these results show that Eya:So are required as *stg-FMW* activators independently of their role as *hth* transcriptional repressors.

Since the RD nuclear protein Dac has also been found as part of the Eya/So complex [52] we tested if Dac also played a role in *stg* transcriptional regulation. In clones of a *dac-*null allele (*dac^3^*) the expression of *stg-FMW* remained unaltered (S5D Fig.), indicating that Dac is not a partner of Eya:So in the regulation of *stg-FMW* enhancer. This finding further indicates that different Eya:So targets may rely on the formation of different protein complexes.

### Hth sharpens the onset of *stg-FMW* activity

Previous results suggested that Hth was a transcriptional repressor of *stg* [19]. However, as we described before, ectopic expression of Hth delayed the onset of *stg-FMW* activation, but did not block it (Fig. 4A), suggesting that *hth* could be involved in the precise timing of *stg-FMW* expression rather than in repressing it. To test this idea, we generated *hth-*mutant clones of a strong allele *(hth^P2^)* [53]. Since *hth-*clones grow poorly [18,19,54], we gave them a growth advantage by using the Minute technique [55]. In *hth- M+* clones the anterior border of *stg-FMW* expression was shifted anteriorly (Fig. 4B). Therefore *hth* is required for the precise spatio-temporal activation of *stg-FMW*, delaying its initiation. Hth is a transcription factor and its action could be mediated through direct interaction with the *stg-FMW* enhancer. In fact, we identified a potential Hth BS in the *stg-FMW* sequence (Fig. 2C). However, mutation of this site (stg*hth) did not result in changes in *stg-FMW* expression (Fig. 4C and S6C Fig.). Although this result does not rule out a direct Hth-DNA interaction through a non-canonical site on the *stg-FMW* enhancer, it points to an indirect effect. In fact, it has been previously shown that Hth and Ey can form a protein complex *in vivo* [17]. The possibility that Hth affects *stg*-FMW through Ey is explored below.

### So/Eya and Ey/Toy act preferentially through different binding sites *in vivo*

**Fig 5:**
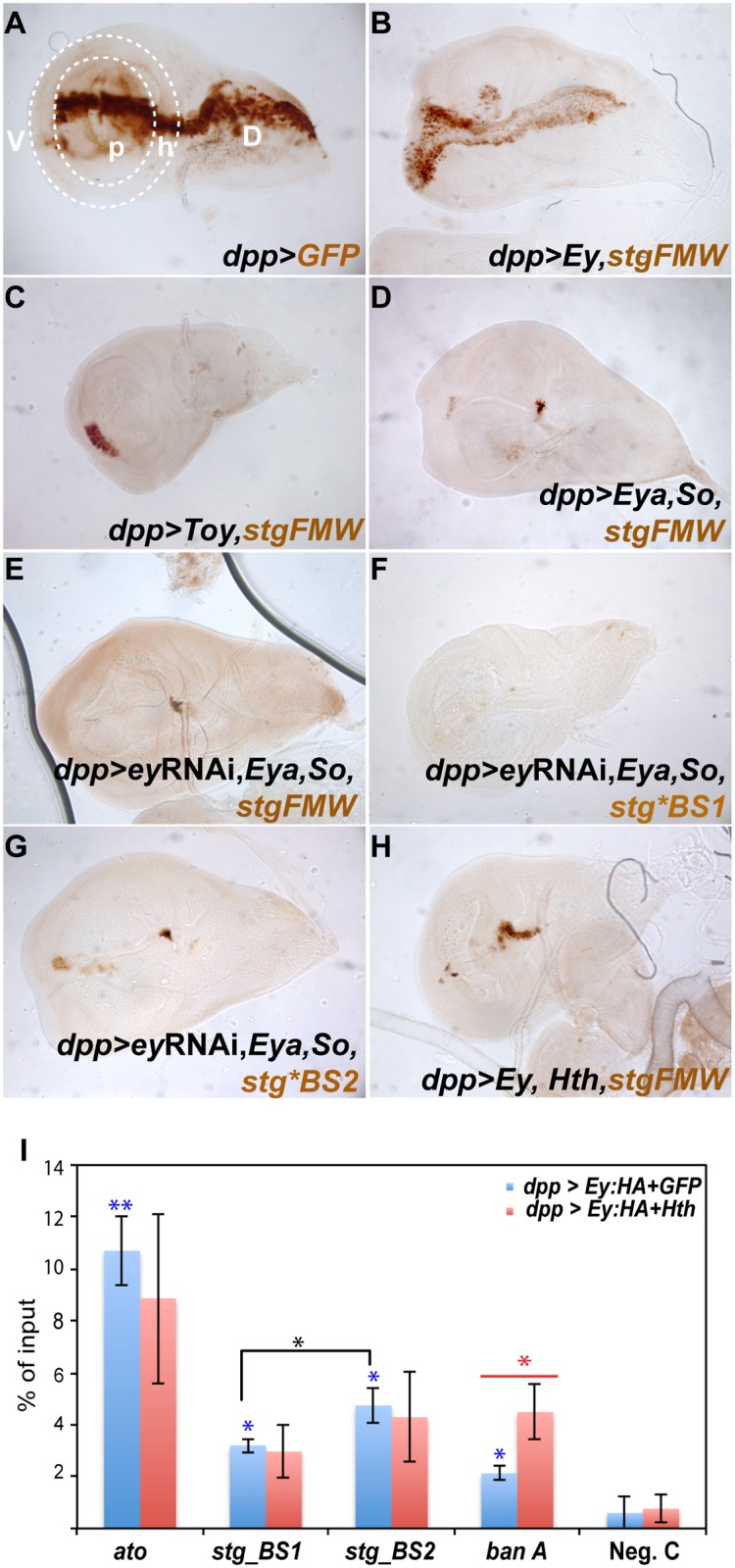
Ey and So regulate *stg-FMW* through different binding sites *in vivo*. (A) L3 wing imaginal disc expressing GFP (brown) in the *dpp*-Gal4 domain, along the anterior-posterior axis. The wing pouch (p) and hinge (h) domains are delimited by the dashed white line. P: pouch, H: hinge, D: dorsal, V: ventral. (B- D) Expression of GFP (brown) driven by *stg*-FMW upon ectopic expression of Ey (B), Toy (C) and Eya/So (D) in the *dpp*-Gal4 domain. (B) Ey is sufficient to promote enhancer activity throughout the Dpp expression domain. (C) Toy is only sufficient to activate *stg*-FMW expression in the ventral hinge region. (D) Ectopic expression of the Eya/So complex can induce enhancer activity in the ventral and dorsal hinge cells of the DPP domain. (E) Activity of *stg-FMW* in discs ectopically expressing Eya/So and *ey* RNAi. Downregulation of Ey, does not affect the ability of Eya/SO to activate the enhancer. (F) Upon mutation of BS1 Eya/So are no longer able to activate *stg*-FMW enhancer. (G) Mutation of BS2 does not affect the ability of Eya/So to activate the *stg-FMW* enhancer even upon downregulation of Ey. (H) Ectopic expression of Hth and Ey in the Dpp domain. The activity of *stg-FMW* is severely compromised when Ey and Hth are co-expressed. (I) ChIP-qPCR experiments show that Ey-HA binds to BS1 and BS2 regions *in vivo* (blue bars). Anti-HA antibodies pulled down *stg-BS2* more efficiently than *stg-BS1*. Sequences from *ato-3’* were used as positive control. In the presence of Hth (red bars) the binding affinity of Ey to *stg-BS1*, *stg-BS2*, and *ato*-3’ is reduced (red bars). Each column shows the averages and standard error of the mean for three independent IPs and real-time PCRs. C: negative control region. Student’s t-test was used for statistical analysis. Blue asterisk: Ey-ChIP recovered chromatin from each region, in *dpp>ey* wing discs, compared to the negative control sample. Black asterisk: Comparison between *stg*-BS1 and *stg*-BS2 regions after Ey ChIP in *dpp>ey* wing discs. Red asterisk: Ey-ChIP recovered chromatin from *dpp>ey* compared to *dpp>ey*, *hth* wing discs. _*_ p ≤ 0,05; _**_ p ≤ 0,005;

Our results show that during eye development Ey/ Toy and So plus Eya are all necessary to activate *stg-FMW*, although in the eye neither the Pax6 genes Ey/Toy or Eya/So are sufficient to do so. Molecularly, mutational analysis of the Pax6 binding sites suggested that Ey and Toy could exert their function through direct binding to BS1 and BS2. To test this hypothesis directly and grasp the molecular interactions underlying *stg*-FMW activity, we performed chromatin immunoprecipitation followed by quantitative real-time PCR (ChIP-qPCR) experiments. We used ectopic gene expression in wing discs as they can be used as a “blank slate” where to assess the functional consequences of expressing RD genes, including Ey. In addition, since in the wing disc Hth expression is restricted to the hinge, we bypass the potential repressor effect of Hth on Ey activity in most of the disc. To drive gene expression we used the *dpp*-GAL4 line, which is expressed in a stripe that bisects the wing disc along its anterior-posterior (A/P) axis (Fig. 5A). *dpp*-GAL4-driven Ey expression (*dpp>ey)* was sufficient to promote activation of the enhancer throughout the Dpp expression domain (Fig. 5B and S7 Fig.), in agreement with the potent eye-inducing ability of Ey [56,57]. In contrast, Toy was only able to induce expression from *stg-FMW* in a small subset of cells in the ventral hinge region (Fig. 5C). This suggests that although in the eye imaginal disc both Ey and Toy have the ability to promote enhancer activity, Ey is a stronger regulator of *stg-FMW* than Toy. We next analyzed the *in vivo* binding of Ey to *stg*-FMW by ChIP-qPCR in *dpp>ey* wing discs. We designed primers so that we could detect binding to region 1 (*stg-BS1*) or region 2 (*stg-BS2*). As positive control we used a region in the *ato-3′* enhancer known to be bound by Ey [24] (Fig. 5I). As expected, we detected a high enrichment of Ey at *ato-3’* relative to our negative control (Fig. 5I). ChIP-qPCR analysis showed that Ey binds to both BS1 and BS2, reinforcing the results described above showing that both sites are used *in vivo*. We consistently recovered higher amounts of chromatin from BS2 than from BS1, suggesting that Ey’s binding affinity towards BS2 region is higher (Fig. 5I), and that this site might be preferentially used by Ey *in vivo*.

Our previous experiments showed a requirement for the Eya:So complex in *stg-FMW* activation, and identified a putative So binding site on region BS1. Ectopic assays in the wing showed that co-expression of Eya and So (*dpp>Eya*,*So)* was able to activate the enhancer in a subset of hinge cells located along the A/P boundary (Fig. 5D and S7 Fig.). However, ectopic expression of So, alone or together with Dac, was not sufficient to activate *stg-FMW*. This observation supports the existence of an Eya:So complex within the precursor domain, whose targets are distinct and independent of the Dac:Eya:So complex. Eya and So can act as transcriptional regulators of Ey [29,49] and it could be argued that the observed *stg*-FMW activity might be indirect and due to Ey up-regulation. To test this point, we checked if ectopic expression of Eya:So in the Dpp domain induced Ey expression. Although ectopic Ey expression was easily detected in the antennal imaginal disc, we systematically failed to detect Ey expression in the wing or leg imaginal discs of *dpp>Eya*,*So* larvae (S7D,G Fig.). These results are in agreement with previous observations [16,48,58]. Nevertheless, and to rule out the possibility that undetectable levels of Ey might contribute to the activation of *stg*-FMW upon ectopic Eya:So expression (Fig. 5D), we used an RNAi to knock *ey* expression down when co-expressing Eya and So (*dpp>eyRNAi*,*Eya*,*So*; Fig. 5E). In these conditions, ectopic *stg-FMW* was induced in the same subset of cells as when induced by Eya and So alone (Fig. 5D, E). This shows that, in ectopic assays, the Eya:So complex has the capacity to promote transcription from the enhancer independently of Ey. This finding allowed us to test if BS1, which contains a putative So binding site, was indeed required for the activation of *stg-FMW* by Eya+So. In case this hypothesis were true, mutation of BS1 should preclude *stg*-FMW activation. To test this point, we checked Eya:So’s ability to activate the enhancer upon mutation of BS1 or BS2, when Ey expression was simultaneously attenuated (*dpp>eyRNAi*,*Eya*,*So*). In this background, mutation of BS1 (stg*BS1) prevented Eya+So from activating the enhancer (Fig. 5F). In contrast, when stg*BS2 was used, the pattern and expression levels of the reporter gene upon Eya+So expression were similar to those of wild-type *stg-FMW* (Fig. 5G). These results suggest that Eya:So complex most likely regulates *stg*-FMW activity through binding to *stg*-BS1. To test this hypothesis we performed ChIP-qPCR experiments using an HA-tagged Eya protein (Eya:HA). As Eya lacks a DNA binding domain, its association with DNA would only occur if forming a complex with its DNA-binding partner So [59]. Thus, Eya ChIP can be used as a read-out of Eya:So target DNA binding. In *dpp>Eya*:*HA* wing discs, anti-HA ChIP-qPCR showed enrichment of *stg-BS1* and *stg-BS2*, although only that for BS2 was statistically significant. *ato*-3’, which was again used as positive control, showed also a significant enrichment, as so did the *ban*A enhancer (also included as control; see below), although to a lower extent (S7H Fig.). Taken together, our results show that the Eya:So complex is able to bind BS2 (and likely also BS1). However, Eya:So regulation of *stg*-FMW relies mostly on BS1.

In addition to the positive regulators Toy, Ey, Eya and So, our experiments indicate that Hth contributes to the precision of the onset of *stg*-*FMW* expression, delaying its activation. The fact that a mutation that eliminates the single canonical Hth binding site does not affect the enhancer’s expression suggested that Hth’s action could be indirect, perhaps mediated through its known interaction with Ey [17]. To test this point, we evaluated the ability of Ey to activate *stg-FMW* in the presence of ectopic Hth (Fig. 5H). In the wing imaginal disc, ectopic expression of Hth strongly reduces the ability of Ey to activate transcription of the reporter gene, which can only be detected in spots in hinge cells (compare Fig. 5H with 5B). To address if Hth counteracts Ey positive role on *stg* transcription by preventing Ey’s binding to chromatin, we performed ChIP-qPCR assays in wing discs upon simultaneous expression of an HA-tagged Ey plus Hth (*dpp>HA*:*Ey*,*Hth*) (Fig. 5I, red bars). The amount of Ey bound to chromatin regions *stg-BS1* and *stg-BS2* in *dpp>HA*:*Ey*,*Hth* was slightly reduced compared to *dpp>HA*:*Ey* (Fig. 5I, blue bars). A stronger reduction was observed for *ato-3′*, the activity of which is known to be directly regulated by Ey binding [24,25]. These results show that Hth only moderately hampers Ey binding to its target DNA sites, something that could be happening through a direct Hth:Ey interaction. Additionally, we noted that *banA*, a CRE from the *bantam* gene, a known direct Hth target in the eye is also bound by Ey (Fig. 5I) [18]. In contrast to *stg* and *ato* sequences, we observe a 2-fold enrichment of the *banA* sequence upon ectopic co-expression of Hth and Ey. This is in agreement with the known binding of Hth to *banA* and likely reflects the previously described ability of Hth to interact with Ey [17,18].

## Discussion

Selector genes lie atop organ-specific gene regulatory networks (GRNs), but it is still unclear what is the depth of their connectivity—i.e. whether selectors regulate a first layer of transcription factors that then relay their information, through consecutive layers down onto specific effector genes (those that determine the actual properties of the cells), or if they regulate the expression at all levels of those GRNs, connecting both to transcription factors and effector genes. This control, in any case, is established by their binding to specific CREs and still, in most organogenetic processes, our knowledge of the molecular logic used by selector genes in GRNs to control gene expression is fragmentary.

The *Drosophila* RD gene network is a good example of this. Despite the vast knowledge of its main components and their contribution to eye development, there is not much evidence about the molecular mechanisms that underlie their function. In particular, how interactions among the different RD gene network components take place and contribute to retina development, by acting not only on other components of the network (all transcription factors or nuclear co-factors) but also on effector genes. In this study we have addressed this question by investigating the mechanisms that regulate *stg* transcription in the developing eye.

*stg* codes for the universal phosphatase that triggers the G2-M transition [60]. Upregulation of *stg* expression during L3 is essential for the synchronous exit from mitosis of retinal progenitors, while simultaneously ensures their amplification at the FMW in order to produce a sufficient number of retinal precursors. It therefore works as an effector gene during the progenitor-precursor cell state transition.

We identified the eye-specific *stg* CRE and showed that it contains two conserved Pax6 binding sites. *Drosophila* has two Pax6 paralogues, *toy* and *ey* [16]. The expression of *toy* starts during early embryogenesis and is required for the activation of *ey* transcription in the eye primordium during late embryogenesis. During larval development, both *toy* and *ey* are coexpressed in the undifferentiated cells of the developing eye primordium [16]. However, while loss of *ey* function during larval stages results in smaller or absent eyes [61,62], no function in the eye had been attributed to the larval expression of *toy*. Here we show that both *ey* and *toy* act as positive regulators of the *stg-FMW* CRE, and that in the absence of *ey*, *toy* suffices to maintain *stg-FMW* CRE activity. However, ectopic experiments in the wing show that their activating capacity differs, with Ey proving to be a more efficient activator of *stg* CRE than Toy. This is consistent with a less powerful eye-inducing ability of Toy compared to Ey [16,56,57]. The discrepancy between the functional equivalence of Ey and Toy in the eye and their different eye-inducing ability in ectopic assays could be explained if Ey expression could facilitate the accessibility of Toy to (at least some) Ey targets. This would happen in the eye (where *toy* activates *ey* very early in the development of the eye primordium) but not in the wing, where none of the two Pax6 genes are normally expressed.

Our work shows that Ey is able to bind both BS1 and BS2, but shows higher affinity towards BS2 *in vivo* (Fig. 5I), suggesting that this site might play a key role in the enhancer activity. In agreement, we found that mutation of BS2, although not affecting *stg-FMW* pattern, causes a significant reduction in its expression levels (S6 Fig.). On the other hand, the transcriptional complex Eya:So is able to bind to BS1 and BS2 with similar affinities, but genetic analysis suggests that interaction with BS1 is critical for *stg-FMW* activity (Fig. 5E-G). However, while this analysis derives from wing disc assays, the mechanism of action in the eye disc might be more complex. While in the wing disc Ey shows a superior *stg-FMW* induction, in the eye removing Eya:So results in loss of enhancer’s activity, despite the fact that Ey and Toy expression remain. This suggests a model in which Toy/Ey and Eya:So cooperate to activate *stg-FMW* enhancer. A similar cooperation between Ey and So has been recently described for the activation of *ato* CREs [24,25], which are also active anterior to the MF with a pattern similar to that of *stg-FMW* [24–26].

The picture that emerges is that of a feed-forward loop, in which Pax6 genes activate Eya and So expression and then Pax6 and Eya:So control *stg-FMW* through direct binding. The engagement of Ey/Pax6 and Eya:So in a positive feed-forward loop has also been reported for the activation of *dac*, even though in this case two separate enhancer elements are involved [33]. Therefore, a similar gene regulatory motif involving Ey and Eya:So operates to control the expression of transcription factors and *stg*, this latter an effector gene. This may be a general feature of the gene networks where Pax6 proteins participate. For example, during the development of the vertebrate eye lens, Pax6 and c-Maf are similarly engaged in a positive feed-forward loop to activate the expression of crystallin genes [63]. This suggests that synergistic interactions among transcription factors within the same GRN determine the specificity of their recruitment to cell type-specific CREs.

**Fig 6:**
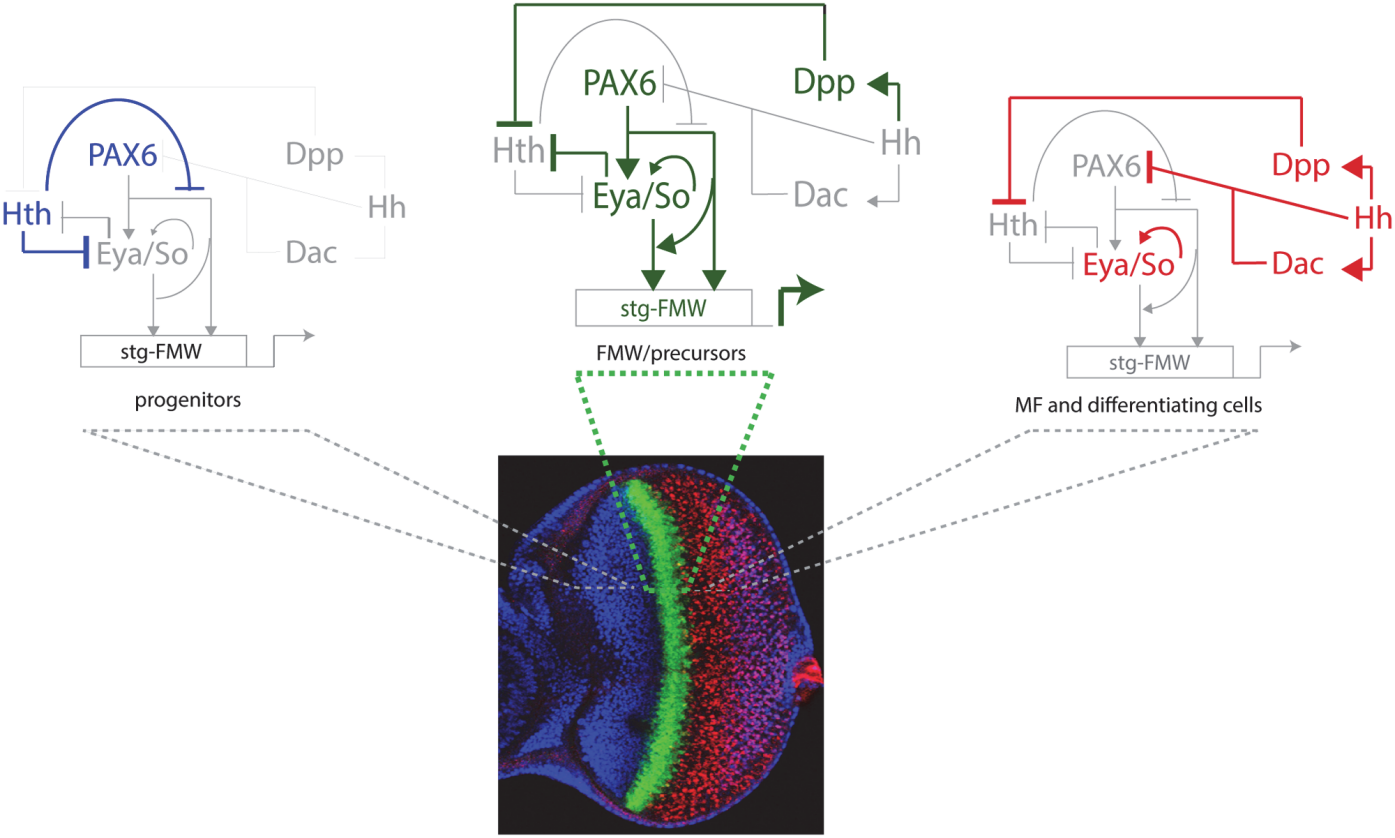
The logic of the eye GRN ensures the pulse activation of the *stg-FMW* CRE in a narrow stripe. Activation requires the contribution of Ey, Toy (collectively referred to as Pax6 in the figure) and the Eya/So complex. In progenitors, Hth expression prevents the activation of *stg-FMW* by repressing Eya/So expression [17] and by reducing the binding and transcriptional activity of Ey (this work). Dpp, acting at long range from its site of production at the MF, represses Hth, thereby alleviating its double repression and allowing the sharp activation of *stg* coincidentally with the upregulation of Eya and So, which engage in an autoregulatory loop [78] in precursor cells. This loop makes their expression independent of Ey. In the eye, both Ey and Eya/So are required for *stg* CRE activation. This is represented by an arrow from Ey to Eya/So that would permit this latter genes to act as transcriptional activators together if Ey is present. At the MF and posterior to it, shorter range repression of Ey by Hh [79] results in the turning off of *stg-FMW*. In addition, after the MF So and Eya are required to keep the enhancer off. A similar concept, the switch of So from an activator to a repressor role at and after the MF, has been recently reported [29] and is in agreement with work showing that So plays essential roles during eye development acting as either transcriptional activator or transcriptional repressor [80].

A key step towards the activation of *stg*-FMW is the repression of Hth, which is mediated by Dpp and Hh [19]. Hth interferes with the coherent feed-forward loop formed by Ey and Eya:So (Fig. 6) at two points: First, Hth moderately hampers Ey binding to *stg*-CRE, something that could contribute to the temporal shifts that this enhancer suffers upon manipulating *hth* function (this work). This could happen through a direct Hth-Ey physical interaction [17]. And second, Hth also acts as a transcriptional repressor of Eya [17]. The resulting GRN allows integration of extracellular signals with tissue specification resulting in a short pulse of *stg* transcription as soon as MF-produced Dpp represses Hth. This pulse is thus made coincidental with the transition from progenitors into precursors (Fig. 6). The need of both Ey and Eya/So inputs for the enhancer’s activation acts as a molecular coincidence detector that ensures that the enhancer will only be active when the regulatory state of the cell is “correct”, avoiding spurious *stg* activation.

Our results also point to a role for *hth* regulation as a precision mechanism, acting to guarantee a sudden, rather than gradual, activation of *stg*. It is through *hth* regulation that the system integrates the extracellular cues with the activity of the selector genes. This mechanism ensures coupling of growth with tissue specification. That *hth* and its vertebrate homologues may play a similar role in Ey/Pax6-regulated processes than the one we have described for *stg* CRE is a tantalizing hypothesis that needs to be investigated. Interestingly, loss of function of Hth does not suffice for enhancer activation in all cells of the anterior domain (Fig. 4). This seems to indicate that additional factors or signaling inputs contribute to *stg-FMW* activation. Dpp and Hh signaling are the obvious candidates. However, ectopic activation of either pathway does not change *stg*-FMW activity in progenitor cells (S8 Fig.) Altogether our data suggests the existence of still unknown anterior factors/signaling inputs that contribute to the regulation of *stg*-FMW expression onset.

The role of Pax6 genes in cancer development appears to be linked with their function during organ development. They act as oncogenes in organs where their expression correlates with the maintenance of the progenitor state, as is the case of the retina and pancreas [reviewed in 2]. In both organs, the maintenance of Pax6 expression during adult stages associates with a failure to undergo differentiation and to tumor development. In contrast, *cdc25* is commonly up-regulated in tumors, as expected from a mitotic gene, but this up-regulation is not tumor type-specific [60]. Our results raise the possibility that Pax6 genes may regulate cell cycle genes in collaboration with Eya/Six proteins also during vertebrate organogenesis, something that might be linked with their oncogenic potential.

## Materials and Methods

### Genotypes and genetic manipulations

The following fly stocks were used: w^*1118*^, *stg* ^*hwy*^ [22], w; FRT82B*hthP2*/TM6B [64], *w; dac* ^*3*^ *FRT40A/ CyO* [65], w; *eyaE8* FRT40 [66,67], UAS-Toy [16], *UAS-eya*, *UAS-so* [24], UAS-*so*, UAS-*dac* (kindly provided by F. Pignoni), *UAS-soRNAi (*VDRC *8950); UAS-eyaRNAi* (VDRC 43911); *UAS-hthRNAi* (VDRC 12764); *UAS-eyRNAi* (VDRC 42845) *and UAS-toyRNAi* (VDRC 15919), *ey* ^*2*^ (Bloomington Stock Center) [61]. Standard genetic techniques were used to introduce *stg-FMW* reporters in the different genetic backgrounds. All crosses were kept at 25°C unless otherwise stated. Cells mutant for *hth* ^*P2*^ were recovered using the Minute technique [55]. The fly strain *yw*,*hs*FLP; FRT82B*hs*CD2, *y+M*/TM2 was used. Mutant tissue was identified by the absence of CD2 staining. Clones were induced between 24 and 48 h or 48 and 72 h after egg laying (AEL) by a 45′ heat-shock (hs) at 37°C. The Flip-Out method [68] was used to induce gain of function clones. The line *yw*,*hs*FLP, *act>hs*CD2>Gal4 was used. Clones were generated at 36–60 h AEL, by a 20′ hs at 35.5°C. Flies were kept at 25°C, except when UAS-RNAi lines were used, in which case they were transferred to 29°C after hs. *Dpp-Gal4/TM6B* (FBti0002123) *and Dpp-Gal4*,*UAS-GFP/ MKRS* (kindly provided by M. Dominguez) were used to ectopically express RD genes in the wing imaginal disc.

### Reporter assays and binding site identification

The FlyC31 system was used to generate all transgenic lines used in this study. Transgenes were inserted in either 2L (22A) or 3R(86FA) attP sites [69]. Insertions on either landing site yielded similar results. Two reporter vectors were used for assaying enhancer activity: pRVV54 that uses nuclear *lacZ* as reporter [70], and pBPUwdGFP which uses destabilized GFP as reporter [71]. *stg-FMW* was cloned into pBPGUw [72] vector to generate *stgFMW*-Gal4 line. Overlapping DNA fragments, covering >60 Kb of *stg* locus were amplified by PCR and introduced into either pBPUwdGFP or pRVV54 using the Gateway System. Mutant versions of *stg-FMW* were cloned into pBPUwdGFP and inserted in 2L (22A) and 3R(86A) sites, as wild-type versions of the enhancer. The megaprimer method was used to generate mutations on putative Ey and Hth binding sites [73]. Primers delimiting *stg-FMW* enhancer sequence and used for enhancer mutagenesis are listed in S1 Table.

### Immunohistochemistry

Imaginal discs were dissected and fixed according to standard protocols. Primary antibodies used were guinea-pig anti-Hth [74], rabbit anti-PH3 (Sigma), rabbit anti β-galactosidase (Cappel), mouse anti β-galactosidase (Sigma), mouse anti-CD2 (Serotec), rabbit anti-GFP (Molecular Probes), mouse anti-Ey (Clements et al., 2008) and rabbit anti-cyclin B [75]. Mouse anti-Eya, rat anti-ELAV (7E8A10), and mouse anti-cyclin B were from Developmental Studies Hybridoma Bank (Iowa University). Fluorescently labeled secondary antibodies were from Molecular Probes. Anti-mouse-HRP (Sigma) was used for immunoperoxidase staining. Digoxigenin labelled *stg* RNA probe was produced from cDNA clone LD47579 (BDGP). ImageJ was used to quantify pixel intensities (http://imagej.nih.gov/ij/).

### Mapping of *stg* ^*hwy*^ mutation

A PCR based approach was used to map and characterize, at the molecular level, the nature of *stg* ^*hwy*^ allele. Several primer combinations spanning the genomic region Chr3R: 25.081.410 to Chr3R: 25.141.369 (Drosophila Genome Release 5) were used to amplify fragments of approximately 5 Kb of DNA from control (*w* ^*1118*^) and *stg* ^*hwy*^ flies. An insertion was detected between genomic coordinates Chr3R: 25114731 and ChR3R: 25115810. Primers flanking and within this region were employed to amplify and sequence *stg* ^*hwy*^ DNA.

### ChIP q-PCR

Wing imaginal discs from wandering L3 larvae of the following genotypes, *dpp>Ey-*HA, GFP; *dpp> Ey-*HA, *Hth-*GFP and *dpp> Eya*:HA, GFP were used for Chip-qPCR analysis. Chromatin was prepared essentially as described in Estella et al. [76]. 30 μg of soluble chromatin, with a size average of 200 bp, was incubated with 3 μg of rabbit anti-HA antibody (AbCam). Ey:HA and Eya:HA bound chromatin complexes were pulled down with protein G magnetic beads (Invitrogen) according to Sandmann et al. [77]. Chromatin was eluted with 100 mM NaHCO_3_. To reverse crosslinking, samples were incubated overnight at 65°C after the addition of 160 mM NaCl. DNA was purified via phenol-chloroform extraction and ethanol precipitation. The PCRs were performed on 1:50 dilutions of the ChIP and input samples. Primers were designed to specifically amplify regions BS1 and BS2, which are 100 bps apart. Primers on *ato*-3’ enhancer were used as positive control [24]. Primers used are described in S1 Table.

## Supporting Information

S1 Fig Sequence of the *stg* ^*hwy*^ allele. (A) Bases shown in black belong to *stg* genomic sequence, while bases shown in red are from the gypsy transposon. (B) Representative L3 eye imaginal disc showing the pattern of GFP driven by the *stg-VS* enhancer fragment.
(TIF)

S2 Fig Developmental expression of *stg*-FMW enhancer recapitulates *stg m*RNA expression. (A-C) Expression of *stg* mRNA at different stages of eye imaginal disc development. Representative discs from late second (A, late-L2), mid third (B, mid-L3) and late third (C, late-L3) larval stages are shown. (D- F) Eye-antennal discs of *stg*-FMW larvae at the second (C, L2), early-L3 (D) and mid-L3 (F) larval stages, stained for GFP, Rhodamine-Phalloidin (Actin), which outlines cell profiles, and the photoreceptor marker Elav. (E, F). No *stg* mRNA or *stg*-FMW expression is detected in L2 discs (A, D) before the onset of differentiation (i.e. before MF onset). The position of the MF is indicated by the dashed white line. Anterior is to the left.
(TIF)

S3 Fig Identification of Ey Binding sites. Representation of the fly Ey PWM used in this study, and the sequences used to generate the matrix. Sequences are derived from *so*, *ato*, *optix*, *eya*, *shf*, and *vha* enhancers.
(TIF)

S4 Fig Expression of *stg* mRNA is not affected in *ey* ^*2*^ mutants. (A—E) *In situ* hybridization of *stg* mRNA on *ey* ^*2*^ homozygous discs shows that the levels and expression pattern of *stg* are not significantly affected, despite the growth defect and abnormal MF progression. The solid white line depicts the MF in *ey* ^*2*^ and the white dashed line represents the position where the MF should be if progression was uniform in dorsal and ventral domains. (B, C) Higher magnification images of the discs in (A). Representative *ey* ^*2*^ eye imaginal discs are shown. (F-G) **The activity of *stg*-FMW enhancer is not affected upon *ey* mutation**. Eye-antennal imaginal discs mutant for ey^2^ showing expression of GFP driven by *stg*-FMW. GFP expression (green) is not affected despite the evident irregular progression of the MF. Eya is shown in red. (H-K) **One Pax6 BS suffices for enhancer activity**. Eye imaginal discs containing clones of *ey* RNAi (H, I) and *toy* RNAi (J, K) in the presence of either stg-BS1* or stg-BS2* mutant *stg-FMW* enhancer. Clones are marked by the absence of CD2 (red) and outlined. GFP expression driven by either stg-BS1* or stg-BS2* is shown in green. In none of these combinations, GFP expression is affected, indicating that Ey and Toy do not show functional preference for either BS1 or BS2.
(TIF)

S5 Fig Eya and So regulate *stg* expression in retina precursor cells. (A) Eya-mutant cells fail to upregulate *stg* mRNA expression. *In situ* hybridization against *stg* mRNA in *eya* ^*E8*^ mutant cells. Clones are marked by the absence of Eya (brown). Mutant cells are outlined. (B) Clones of *so RNAi* are labeled by the absence of CD2 (red) and show that expression of *stg-FMW* (green) is repressed anterior to the MF. Hth, shown in blue, is maintained in *so-*mutant cells. (C) MARCM *eya^-^hth^-^* double mutant clones (GFP: *eya^E8^;hth-RNAi*) stained for Ey (red). Ey is maintained in *eya^-^hth^-^* cells. (D) Clones of loss of function of *dac*, labeled by the absence of β-gal (red). In *dac* ^*3*^ mutant cells expression of *stg-FMW* (green) is not affected. Hth is shown in blue.
(TIF)

S6 Fig Representative third instar eye imaginal discs carrying the wild type version of *stg-FMW* enhancer (A), and upon mutation of Ey/Pax stg*BS1 (B) stg*BS2 (C) and Hth core BSs, stg*Hth (D). (E) Quantification of GFP expression levels driven by wild type and mutant versions of *stg*-FMW enhancer. At least eight eye imaginal discs were analyzed per genotype. Student’s t-test was applied for statistical analysis. _**_ p≤ 0,005.
(TIF)

S7 Fig Expression of Ey and Eya:So in the Dpp domain induces the expression of other members of the RD network. Ectopic expression of Ey (A—C) leads to upregulation of Eya (B) and Dac (C) expression along the A/P boundary in L3 wing imaginal discs. (A) Ey immunoreactivity in a *dpp>Ey* disc. Upon ectopic expression of Eya:So up-regulation of Ey expression is only detected in the antenna imaginal disc (G). Expression of Ey is not detected in the leg or wing imaginal discs (D). Ectopic expression of Eya:So (E) induces upregulation of Dac expression along the A/P border (F). In (G) the white arrow indicates the normal domain of Ey expression in the anterior region of the eye field and in the brain; double arrow indicates ectopic expression of Ey in the antenna imaginal disc. (H) Eya:So binds to *stg*-FMW *in vivo*. Eya:HA was used to precipitate chromatin from *dpp>Eya-HA* wing imaginal discs. Sequence from *ato-3’* enhancer *(“ato”)* was used as positive control. The graph represents the percentage of signal obtained relative to input chromatin. The average and standard deviation in two independent ChIP experiments are shown. A significant enrichment for Eya:HA was observed for the positive control (“*ato”)*, *stg-BS2* and *banA*, but not for the negative control (“C”). Student’s t-test was used for statistical analysis. _**_ p ≤ 0,005.
(TIF)

S8 Fig Activation of the *Dpp* or the *Hh* pathway does not lead to precocious activation of *stg-FMW*. Clones of gain of function of an activated form of *thickveins* (*tkv* ^QD^) (A-C) and *Cubitus interruptus* (*ci*) (D) showing that the Dpp and Hh signalling pathways do not suffice for activation of *stg*-FMW in other domains of the eye imaginal disc. Clones are labelled by the absence of CD2 (magenta). The activity of *stg*-FMW is detected by the expression of GFP (green). Clones are outlined. Anterior is to the left.
(TIF)

S1 Table List of oligonucleotide sequences used in this study. (PDF)
