# Supplementary figures and images for: Eye Selector Logic for a Coordinated Cell Cycle Exit
Source: PLoS Genet. 2015 Feb 19;11(2):e1004981. doi: 10.1371/journal.pgen.1004981 (PMC4335009; doi:10.1371/journal.pgen.1004981)

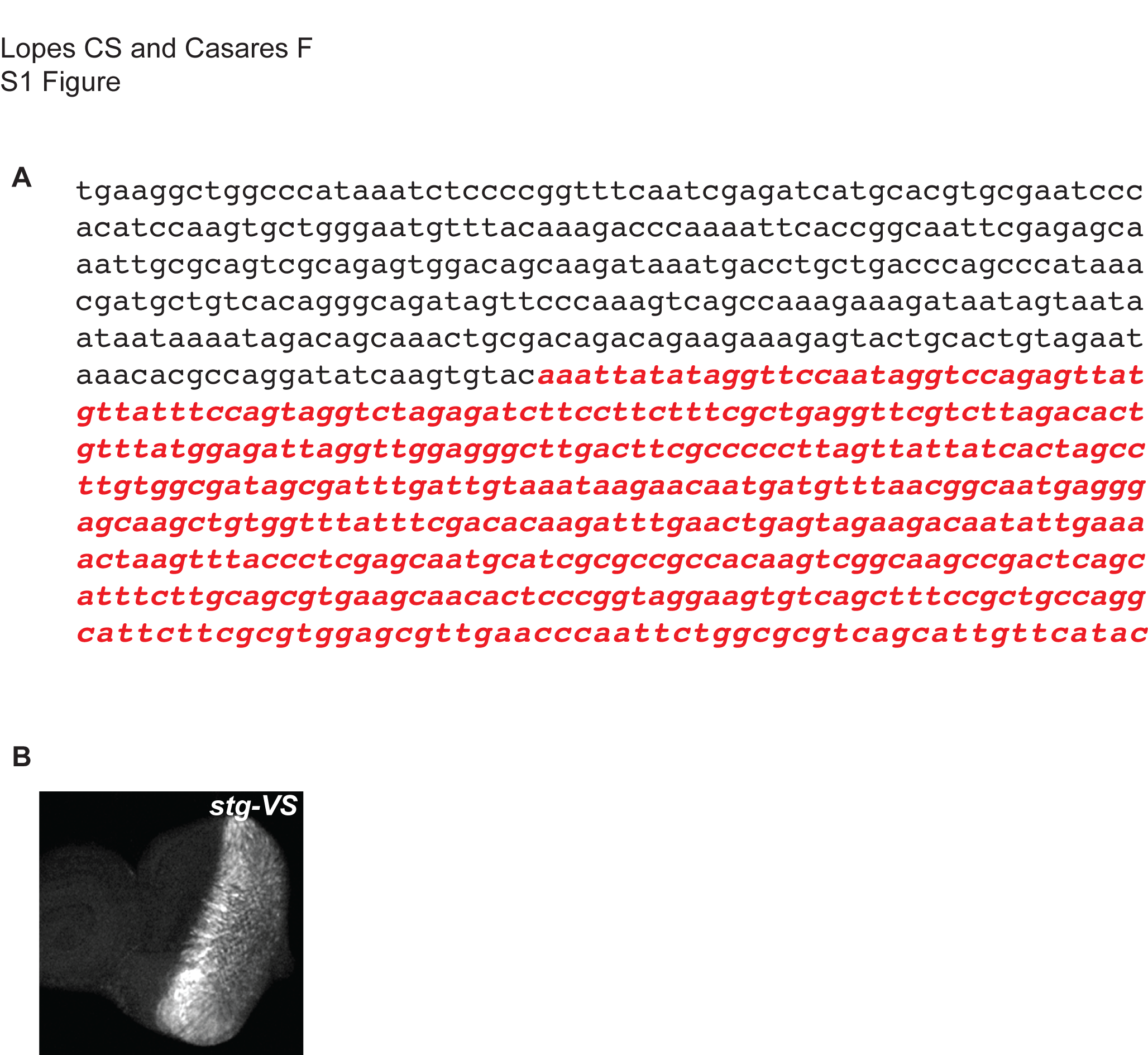

Supplement: S1 Fig — (A) Bases shown in black belong to stg genomic sequence, while bases shown in red are from the gypsy transposon. (B) Representative L3 eye imaginal disc showing the pattern of GFP driven by the stg-VS enhancer fragment. (TIF) [file pgen.1004981.s001.tif]

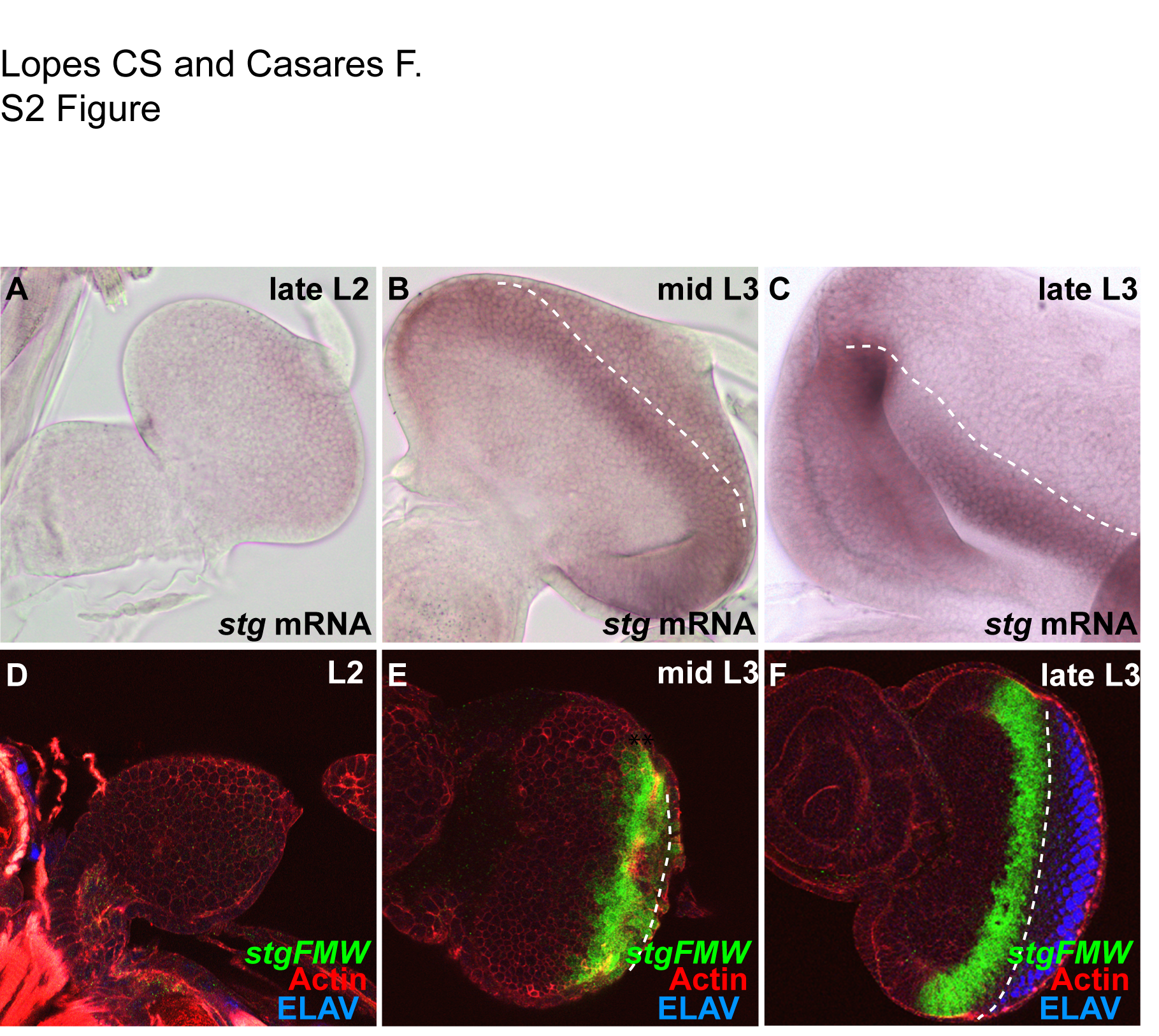

Supplement: S2 Fig — (A-C) Expression of stg mRNA at different stages of eye imaginal disc development. Representative discs from late second (A, late-L2), mid third (B, mid-L3) and late third (C, late-L3) larval stages are shown. (D- F) Eye-antennal discs of stg-FMW larvae at the second (C, L2), early-L3 (D) and mid-L3 (F) larval stages, stained for GFP, Rhodamine-Phalloidin (Actin), which outlines cell profiles, and the photoreceptor marker Elav. (E, F). No stg mRNA or stg-FMW expression is detected in L2 discs (A, D) before the onset of differentiation (i.e. before MF onset). The position of the MF is indicated by the dashed white line. Anterior is to the left. (TIF) [file pgen.1004981.s002.tif]

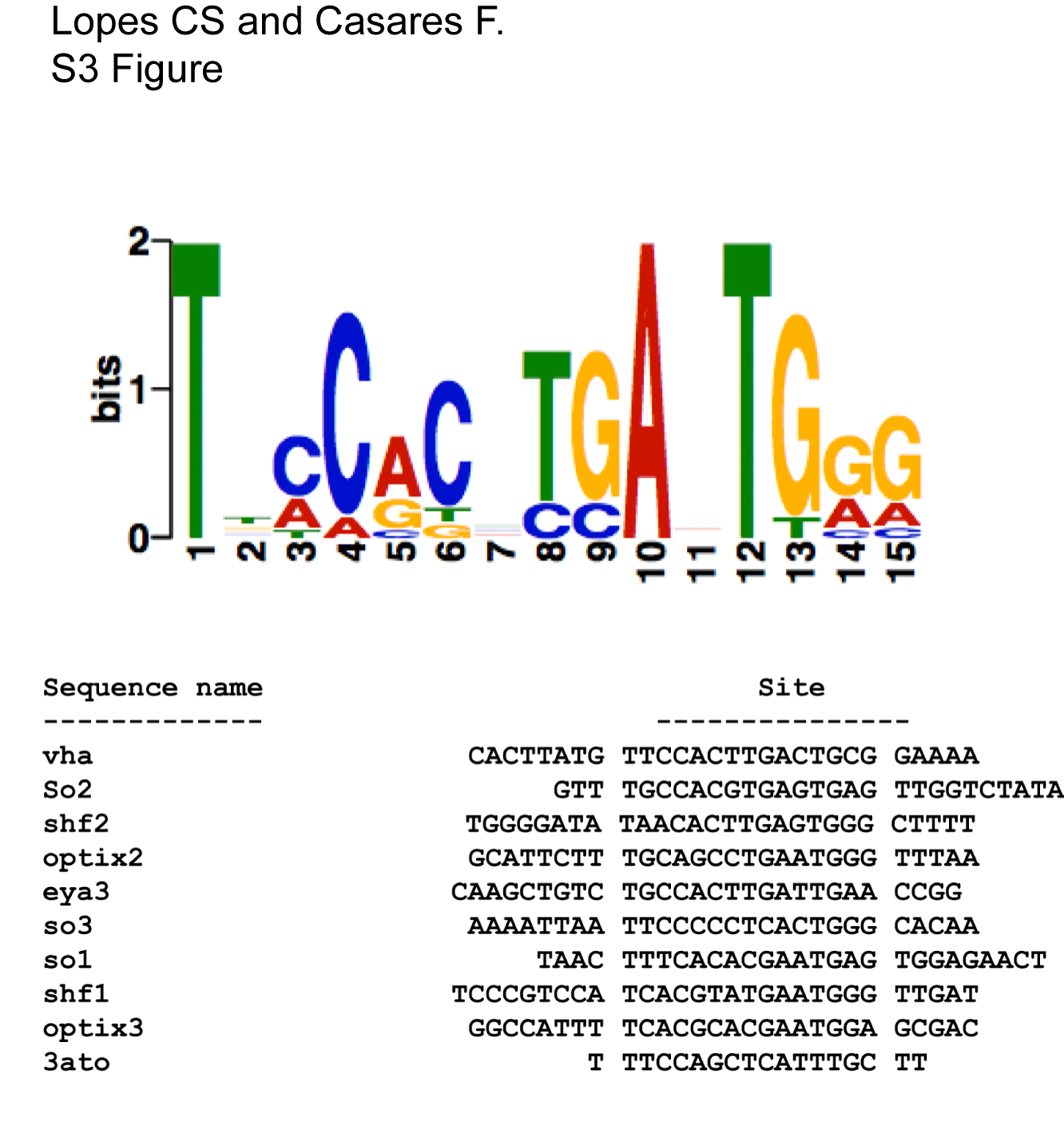

Supplement: S3 Fig — Representation of the fly Ey PWM used in this study, and the sequences used to generate the matrix. Sequences are derived from so, ato, optix, eya, shf, and vha enhancers. (TIF) [file pgen.1004981.s003.tif]

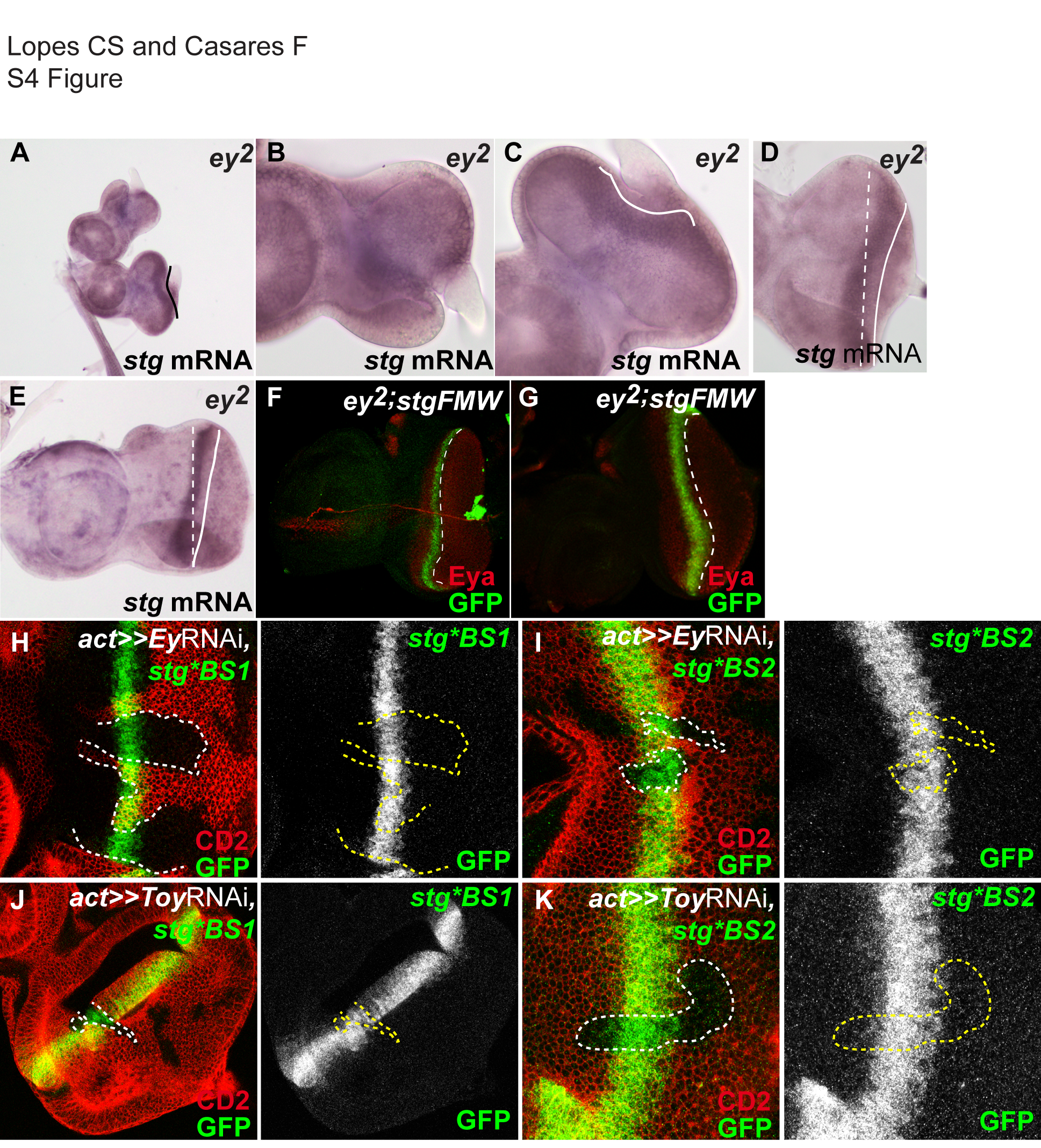

Supplement: S4 Fig — (A—E) In situ hybridization of stg mRNA on ey 2 homozygous discs shows that the levels and expression pattern of stg are not significantly affected, despite the growth defect and abnormal MF progression. The solid white line depicts the MF in ey 2 and the white dashed line represents the position where the MF should be if progression was uniform in dorsal and ventral domains. (B, C) Higher magnification images of the discs in (A). Representative ey 2 eye imaginal discs are shown. (F-G) The activity of stg-FMW enhancer is not affected upon ey mutation. Eye-antennal imaginal discs mutant for ey2 showing expression of GFP driven by stg-FMW. GFP expression (green) is not affected despite the evident irregular progression of the MF. Eya is shown in red. (H-K) One Pax6 BS suffices for enhancer activity. Eye imaginal discs containing clones of ey RNAi (H, I) and toy RNAi (J, K) in the presence of either stg-BS1* or stg-BS2* mutant stg-FMW enhancer. Clones are marked by the absence of CD2 (red) and outlined. GFP expression driven by either stg-BS1* or stg-BS2* is shown in green. In none of these combinations, GFP expression is affected, indicating that Ey and Toy do not show functional preference for either BS1 or BS2. (TIF) [file pgen.1004981.s004.tif]

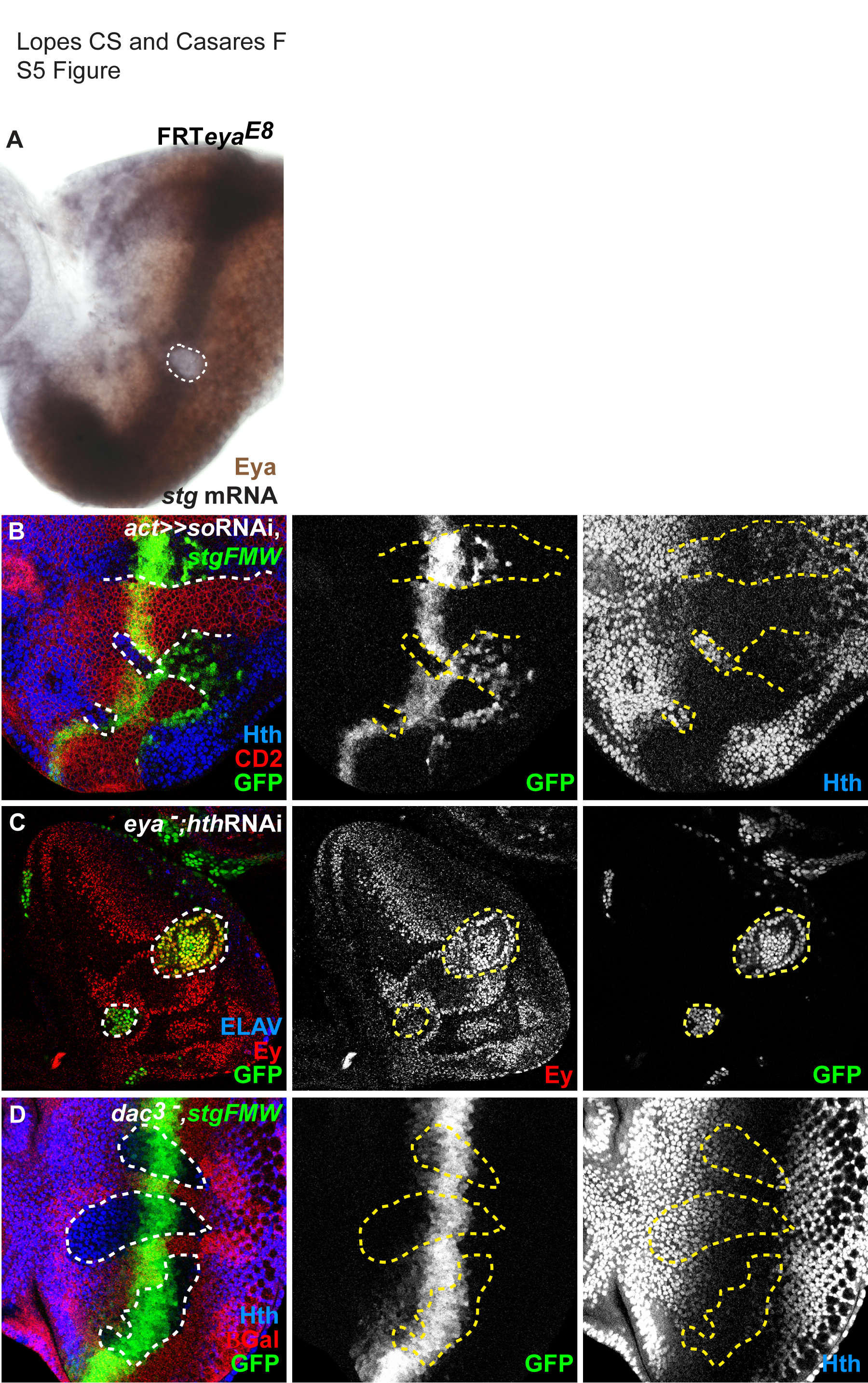

Supplement: S5 Fig — (A) Eya-mutant cells fail to upregulate stg mRNA expression. In situ hybridization against stg mRNA in eya E8 mutant cells. Clones are marked by the absence of Eya (brown). Mutant cells are outlined. (B) Clones of so RNAi are labeled by the absence of CD2 (red) and show that expression of stg-FMW (green) is repressed anterior to the MF. Hth, shown in blue, is maintained in so-mutant cells. (C) MARCM eya-hth- double mutant clones (GFP: eyaE8;hth-RNAi) stained for Ey (red). Ey is maintained in eya-hth- cells. (D) Clones of loss of function of dac, labeled by the absence of β-gal (red). In dac 3 mutant cells expression of stg-FMW (green) is not affected. Hth is shown in blue. (TIF) [file pgen.1004981.s005.tif]

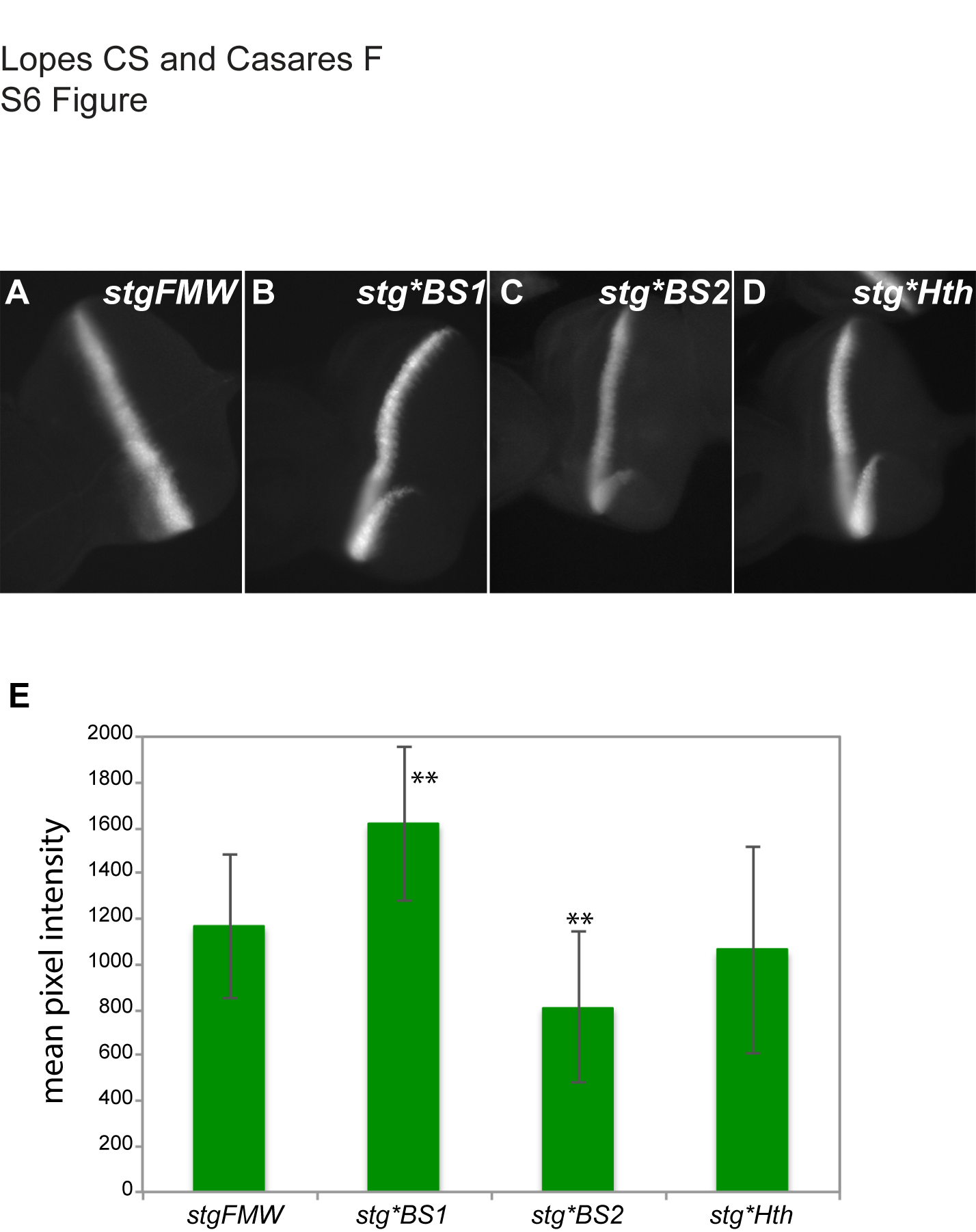

Supplement: S6 Fig — (E) Quantification of GFP expression levels driven by wild type and mutant versions of stg-FMW enhancer. At least eight eye imaginal discs were analyzed per genotype. Student’s t-test was applied for statistical analysis. ** p≤ 0,005. (TIF) [file pgen.1004981.s006.tif]

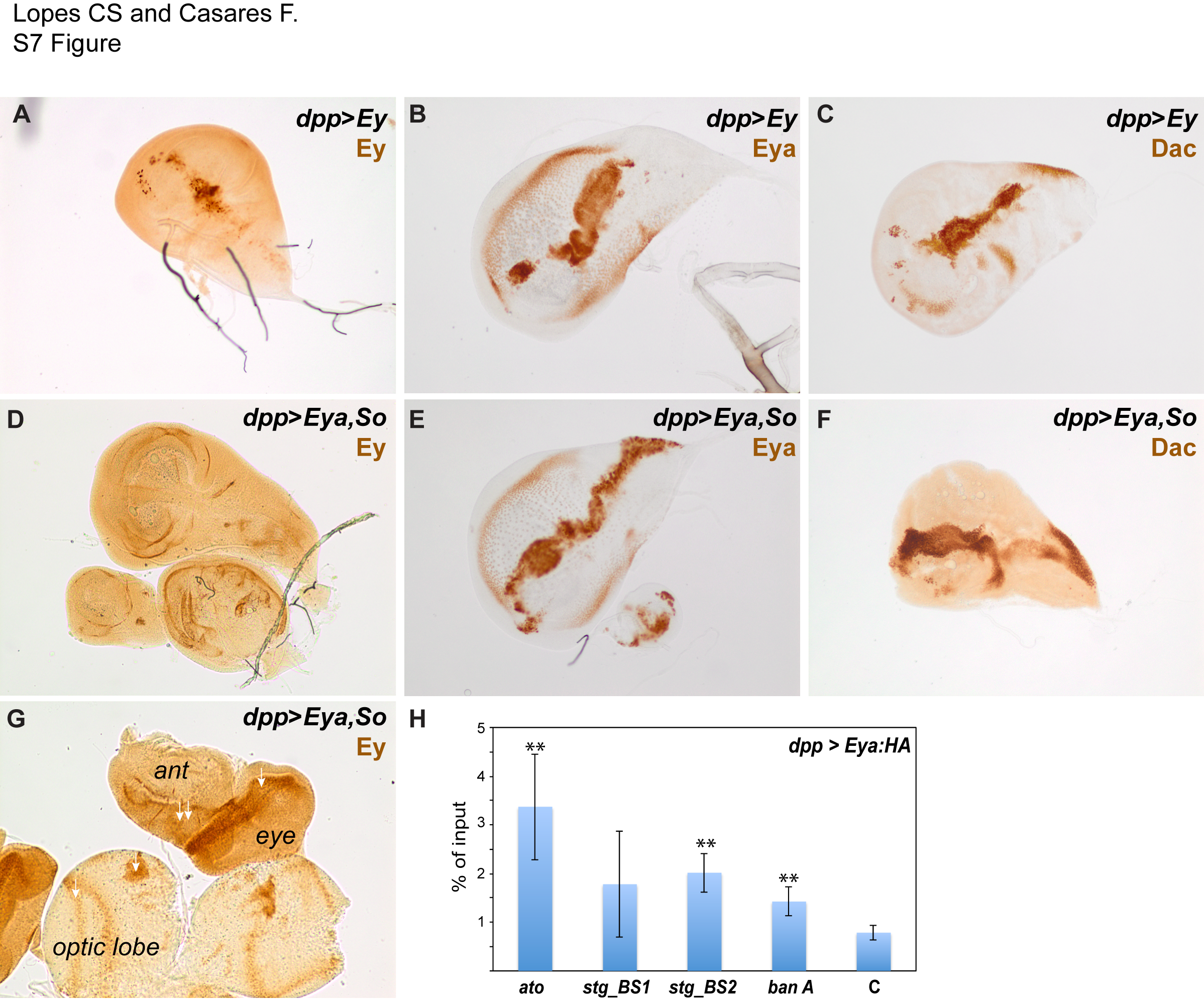

Supplement: S7 Fig — Ectopic expression of Ey (A—C) leads to upregulation of Eya (B) and Dac (C) expression along the A/P boundary in L3 wing imaginal discs. (A) Ey immunoreactivity in a dpp>Ey disc. Upon ectopic expression of Eya:So up-regulation of Ey expression is only detected in the antenna imaginal disc (G). Expression of Ey is not detected in the leg or wing imaginal discs (D). Ectopic expression of Eya:So (E) induces upregulation of Dac expression along the A/P border (F). In (G) the white arrow indicates the normal domain of Ey expression in the anterior region of the eye field and in the brain; double arrow indicates ectopic expression of Ey in the antenna imaginal disc. (H) Eya:So binds to stg-FMW in vivo. Eya:HA was used to precipitate chromatin from dpp>Eya-HA wing imaginal discs. Sequence from ato-3’ enhancer (“ato”) was used as positive control. The graph represents the percentage of signal obtained relative to input chromatin. The average and standard deviation in two independent ChIP experiments are shown. A significant enrichment for Eya:HA was observed for the positive control (“ato”), stg-BS2 and banA, but not for the negative control (“C”). Student’s t-test was used for statistical analysis. ** p ≤ 0,005. (TIF) [file pgen.1004981.s007.tif]

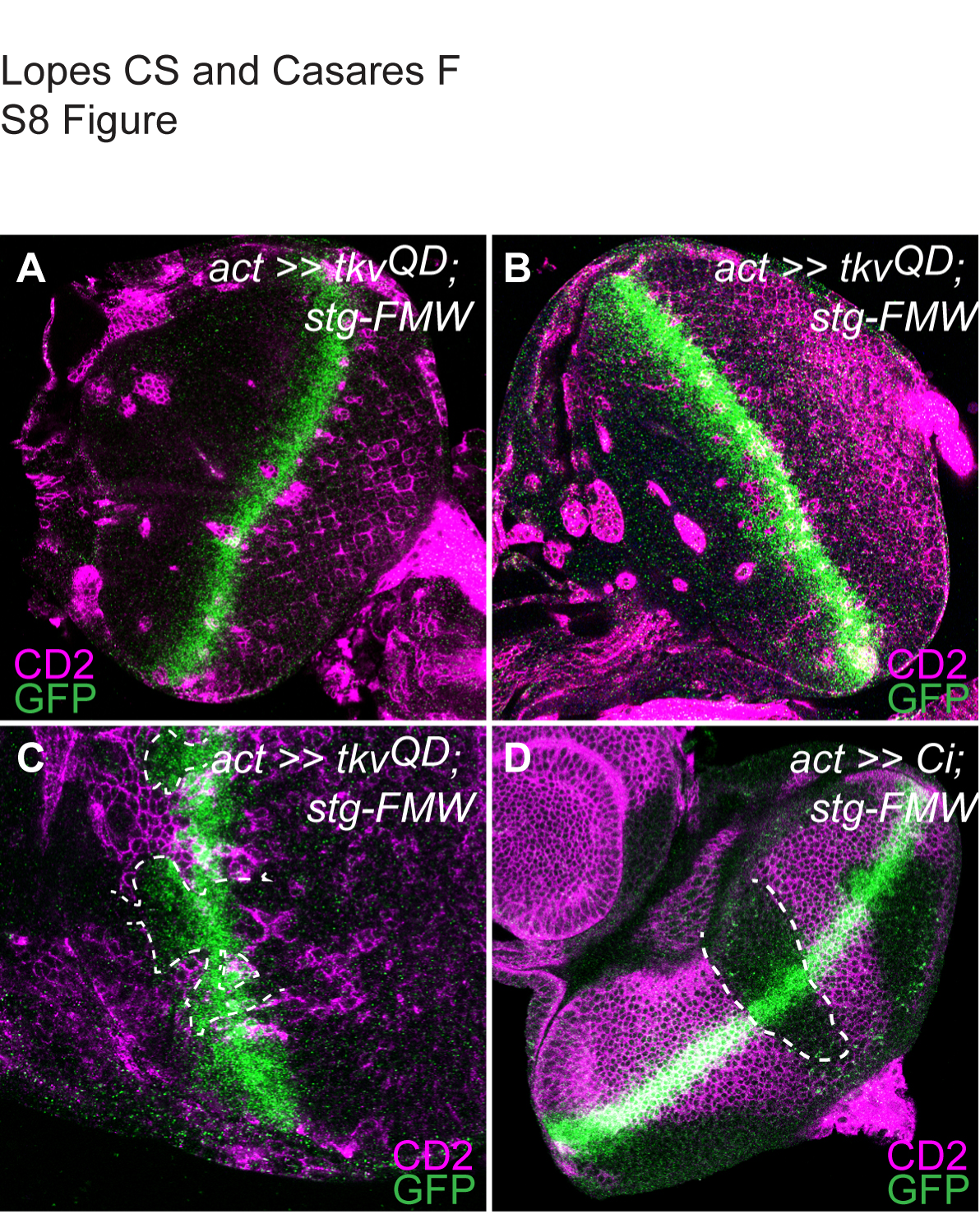

Supplement: S8 Fig — Clones of gain of function of an activated form of thickveins (tkv QD) (A-C) and Cubitus interruptus (ci) (D) showing that the Dpp and Hh signalling pathways do not suffice for activation of stg-FMW in other domains of the eye imaginal disc. Clones are labelled by the absence of CD2 (magenta). The activity of stg-FMW is detected by the expression of GFP (green). Clones are outlined. Anterior is to the left. (TIF) [file pgen.1004981.s008.tif]
