# Supplementary material for: Eye Selector Logic for a Coordinated Cell Cycle Exit
Source: PLoS Genet. 2015 Feb 19;11(2):e1004981. doi: 10.1371/journal.pgen.1004981 (PMC4335009; doi:10.1371/journal.pgen.1004981)
Supplement: S1 Table — (PDF) [file pgen.1004981.s009.pdf]

Table 1: Sequence of oligos used in this work.

**Name**	**Sequence (5’-3’)**
stg 25.081.410 FW	GTAGAATCCTTTCCCCTGCACTT
stg 25.141.500 RW	AACTCTGTACGTACTTTTGCCGTAT
Stg-FMW FW	TATGTGTTTATTACGGGCACCC
Stg-FMW RW	CAGGGACATAAACAGAAATTCG
stg-FMW BS1* RW	GAGGAGGATGGTCAAATGTGAACCATCACCGAGGACAAGGAT
stg-FMW BS2*	TGCTGGTGTGATGTTCTCTTAACTTGCTGTCAGCGTGGCTGCTCC
stg-FMW Hth*	GATTTGGAAGGACGAGGCGTGGCTGCTCCAATTTCATG
stg_25114731	ATGCATGAGCAATTATCGATTCTACG
stg_25115810	GTCATGTTGGCAATCTCGGCTTTCTGC
**Real –time PCR oligos**	
ato_FW	CGGGGCCTGTAATTTTGTAT
ato_RW	GAGACTTTGGTGCCCAATTA
stg_Ey1 FW	CGGCGCGGAATCCTTGAAGT
stg_Ey1 RW	CATATAGGATCGCACAAATTGC
stg_Ey2 FW	TATATGTGGTGCTGGTGTGC
stg_Ey2 RW	AGTGCACTTGAGCAAATCG
banA FW	AATCCAAACGTGCAGACGGC
banA RW	AGCGGTGTCTAAGCACAGCG
C_neg_FW	AGCTAGTTGCCACCTTTCGT
C_neg_RW	ACAATCACTCACTCGCTTGC
